# Gut microbiome-derived indole-3-carboxaldehyde promotes intestinal development via AHR-NRF2 signaling in the early-life of chicks

**DOI:** 10.1186/s40168-025-02289-2

**Published:** 2025-12-16

**Authors:** Yu-Xuan Huang, Zhang-Chao Deng, Ke-Xin Cao, Jia-Cheng Yang, Meng Liu, Ling Zhao, Jin-Shui Zheng, Lv-Hui Sun

**Affiliations:** 1 National Key Laboratory of Agricultural Microbiology, Key Laboratory of Smart Farming Technology for Agricultural Animals, Ministry of Agriculture and Rural Affairs, Hubei Hongshan Laboratory, Frontiers Science Center for Animal Breeding and Sustainable Production, College of Animal Science and Technology, Huazhong Agricultural University, Wuhan, 430070 China; 2 Department of Animal Science, Cornell University, Ithaca, NY 14850 USA

**Keywords:** Intestinal epithelial barrier function, Chicks, *Lactobacillus*, Indole-3-Carboxaldehyde, Indole-3-Carboxylic acid

## Abstract

**Background:**

The development of the small intestine is crucial during early life, with the gut microbiota and microbe-derived metabolites playing key roles in regulating intestinal epithelial barrier function and overall development. However, the underlying mechanism remains unclear. Here, chicks were used to investigate the influences of early-life crosstalk among bacteria, metabolites, and the host on small intestinal development.

**Results:**

We investigated bacterial succession in the small intestine of broiler chicks at four time points during early development. After 3 days post-hatch, Bacillota became the dominant phylum. At the genus level, *Lactobacillus* and *Ligilactobacillus* emerged as the two dominant genera, and their abundance was significantly positively correlated with small intestine weight. Metabolome analysis revealed that indole-3-carboxaldehyde (IAld) is derived from both *L. gallinarum* C2-16–2 (LG) and *L. salivarius* D7-21 (LS). Moreover, we found that IAld can be converted into bioactive indole-3-carboxylic acid (ICA) in animals, which exhibited greater biological activity than IAld in vitro. Further chick feeding trials revealed that both bacteria (LG and LS) and metabolites (IAld and ICA) promoted epithelial barrier function and enhanced antioxidant capacity during early life in chicks. Moreover, both IAld and ICA promoted tight junction protein expression and enhanced antioxidant capacity by activating AHR-NRF2 signaling.

**Conclusions:**

These findings suggest that specific bacterial strains (*L. gallinarum* C2-16–2 and *L. salivarius* D7-21) and metabolites (IAld and ICA) serve as effective promoters of intestinal epithelial barrier function and antioxidant capacity during early intestinal development in chicks

Video Abstract

**Supplementary Information:**

The online version contains supplementary material available at 10.1186/s40168-025-02289-2.

## Background

In the early stages of life, young animals face abrupt physiological changes, such as aerial breathing, thermal regulation and dramatic nutritional shifts. These challenges, coupled with underdeveloped intestines, can limit nutrient digestion and absorption, necessitating efficient adaptation to achieve genetic growth potential. The gut microbiota and its metabolites serve as signalling molecules or substrates that contribute to various functions in animals, including nutrient digestion, feed efficiency, cell proliferation, and immune regulation [1–3]. The microbiota colonizes the gut of animals at birth through vertical transmission from the mother and dietary intake, with the cecum harboring the largest microbial population [4, 5]. Although many studies have employed culture-dependent and culture-independent methods to investigate the gut microbiota in faeces and the cecum, the bacterial communities of the small intestine remain relatively poorly understood [6–8]. Compared with the duodenum, jejunum, and ileum, the small intestine plays a pivotal role in nutrient digestion and absorption and acts as a vital interface for the efficient transfer of nutrients into the bloodstream [9, 10]. Although the absolute abundance of microbes in the small intestine is relatively low, the microbiota and their metabolites are in close proximity to the small intestinal epithelium, a single-cell layer that separates host components from the external environment. The integrity of the gut barrier is maintained by tight junction proteins, including claudins, zona occludens-1 (ZO-1), and occludin. Their expression patterns exhibit remarkable spatial and temporal dynamics throughout gut development, which are essential for epithelial barrier function and promoted by probiotics (such as *Lactobacillus* and *Ligilactobacillus*) and postbiotics [2, 11].

Chickens are among the most widely raised animals worldwide and play a vital role in global food production [12]. In industrially raised broilers, the absence of maternal contact disrupts the natural transmission of microbiota from the hens, making the initial colonization and subsequent maturation of the gut microbiota after hatching highly dependent on external factors, particularly dietary accessibility. Unlike mammals, birds have shorter digestive tracts, and the microbial composition across different segments of the small intestine is relatively similar, further emphasizing the need for a well-established gut microbiota to optimize nutrient absorption and immune function [5, 6]. The early post-hatch period is a critical phase in the life of broiler chickens, marked by rapid physical and functional development of the gastrointestinal tract and a transition from yolk-based to complex dietary nutrition, laying the foundation for their growth and health [13]. However, the mechanisms by which the microbiota and its metabolites influence small intestinal development and chick growth during early life remain unclear.

To fill this gap, we employed a discovery cohort to identify the dominant bacterial communities and metabolites associated with intestinal development during chick growth. A confirmatory cohort was subsequently used to validate the identified bacteria and metabolites and to explore the potential underlying mechanisms. In this study, we used a confirmatory cohort of early-life chicks to investigate how key bacterial taxa and their associated metabolites influence intestinal development. Through integrated bacteriome and metabolome profiling, we evaluated their effects on intestinal weight, tight junction protein expression, circulating lipopolysaccharide (LPS) levels, serum diamine oxidase (DAO) activity, and antioxidant capacity.

## Methods

### Collection of intestinal tissue and content samples

The animal procedures used in this study were approved by the Institutional Animal Care and Use Committee of Huazhong Agricultural University, Wuhan, China (HZAUCH-2024–0011).

A total of 64 chicks were used in the discovery cohort to dissect the landscape of chick gut bacteriome in one hundred 1-day-old male broilers' population that hatched on the same day in a poultry farm (Wuhan, China). And 400 1-day-old male broilers were used in the confirmatory cohort. The chicks were divided into 5 experimental groups (8 cages per group, 10 birds per cage). The broilers were allowed free access to feed and water. The diet was formulated according to the feeding standards of Chinese chickens (NY/T33‐2004, Additional file 1: Table S1). The LG and LS groups received feed supplementation with *L. gallinarum or L. salivarius* (10^11^ cfu/kg) every day, respectively. The IAld and ICA groups received feed supplementation with 100 mg/kg of IAld or ICA, respectively. Whole blood and the intestinal content of three segments (duodenum, jejunum, and ileum) were collected and frozen in liquid nitrogen followed by storage at − 80 °C for further analysis.

### Bacterial 16S rRNA gene sequencing and data analysis

The contents of the duodenum, jejunum, and ileum were pooled for DNA extraction. Microbial DNA extraction was performed according to the protocol described previously [7]. The DNA concentration of the extracted microbial genomes was quantified by Thermo Scientific™ NanoDrop™ (CA, USA). The bacterial V3-V4 region was amplified using the following primers: F, 5’-CCTACGGGNBGCASCAG-3’ and R: 5’-GACTACNVGGGTATCTAATCC-3’. [14]. High throughput sequencing was conducted on the Illumina HiSeq2500 platform using a paired-end 250 bp (PE250) strategy, generating ~ 460 bp amplicons. Clean reads were obtained from sequencing raw data using fastp (v0.22.0) [15]. We assembled quality-filtered reads into amplicon sequence variants (ASVs) using modified Parallel-Meta Suite (PMS) pipeline [16]. Briefly, all unassigned sequences and sequences annotated as mitochondria and chloroplasts were removed. Matrix was rarefied to the same number of counts for downstream analyses. The Alpha diversity, principal coordinate analysis (PCoA) and Non-metric MultiDimensional Scaling (NMDS) based on Bray–Curtis dissimilarity were calculated using the R package vegan [17]. The Linear discriminant analysis Effect Size (LEfSe) was performed based on Galaxy LefSe [18].

### Network analysis, stochasticity of the gut microbiota assembly and Enterotype clustering

Bacterial taxon-taxon co-occurrence networks were constructed using Sparcc correlation and the R package SpiecEasi in different days post hatch (DPH). The false discovery rate (FDR) was used in each network, and FDR > 0.05 was filtered out. The network topological structures were visualized by chiplot (http://chiplot.online) and the network properties and keystone taxa were calculated by ggClusterNet [19]. Between-class analysis (BCA) models were used to assign the samples to the bacterial communities types (enterotypes) [20]. The appropriate number of clusters was determined based on the highest Calinski–Harabasz index.

To calculate the relative importance of determinism and stochasticity in bacterial assembly, a two‐step procedure was applied considering β‐nearest taxon index (βNTI) and Bray–Curtis‐based Raup–Crick Index (RCBray) values, including heterogeneous selection, homogeneous selection, dispersal limitation, homogenizing dispersal, and undominated. The values βNTI and RCBray were calculated using the R package NST [21].

### Bacteria isolation and identification

Digesta samples were taken to an anaerobic workstation (Longyue, Shanghai, China) and tenfold serially diluted with phosphate buffered saline (PBS). Isolates were obtained from the cultivation of the samples by plating 0.1 mL of dilutions from 10^–3^ to 10^–7^ into De Man, Rogosa and Sharpe (MRS) agar, M17 agar and Bifidobacterium selective Medium (BSM).

Dilution plates were observed every 24 h and new colonies were collected at 24, 48, and 72 h. All isolates were serially cultured into new culture media to obtain pure cultures. Cultures were incubated at 37 °C under anaerobic conditions of a gas mixture of 85% N_2_, 10% CO_2_ and 5% H_2_. All the strains were preserved in liquid medium with a 25% glycerol solution at − 80 °C.

Fresh bacterial cultures were divided into two portions, one for gram staining followed by microscopic examination and the other for DNA extraction. DNA extraction was performed according to the manufacturer’s protocol (BL1044A, BioSharp). Briefly, 1 mL of bacterial cells was washed twice with PBS and the bacterial pellet was suspended in 0.2 mL PBS. After incubating with lysozyme and proteinase K, tubes were centrifuged at 14,000 rpm for 1 min and then the supernatant was recovered and purified using a DNA-binding column. The DNA concentration of the extracted bacterial genomes was measured using the fluorometric method with Qubit ® 3.0 Fluorometer (Life Technologies, CA, USA). DNA samples were stored at − 20 °C.

Genomic DNA was identified by analyzing 16S rRNA gene sequences using the primers 27 F and 1492R [22]. Amplicons were identified using the BLAST tool [23] and aligned to the closely related species at the Non-redundant (NR) Database from the National Center for Biotechnology Information (NCBI).

### Phenotypic scoring and ranking of isolates

Readouts from acid tolerance (pH 3), bile-salt tolerance (4% w/v), NaCl tolerance (8% w/v), and antibacterial activity were aligned so that higher values indicate better performance, then robustly standardized within the assay using the median. A composite score was computed as the mean of robust z-values. Strains were ranked by this score (Table S2).

### Bacterial Genome sequencing, assembling, and annotation

Each sample of 0.5 µg genome DNA was prepared following Annoroad® Universal DNA Fragmentase kit V2.0 and Annoroad® Universal DNA Library Prep Kit V2.0 protocols. The DNA libraries were sequenced using a 150 PE run from Illumina NovaSeq 6000 platform. Reads were quality controlled and decontaminated from sequencing artefacts and adapters and merged using fastp (v0.22.0) [15]. Assembling was performed using SPAdes (v3.15.0) [24]and further gene structure and function annotation were done with Prokka (v1.14.5) [25]. Protein sequences were functionally annotated using the eggNOG 5.0 database [26].

### Metabolic profiling of bacterial ferment and chicken serum

For untargeted metabolomics, the hydrophilic metabolites were extracted according to the protocol described previously [27, 28]. Briefly, the serum samples or bacterial fermentation samples (100 µL) were thawed on ice and 400 μL HPLC-grade methanol:water (v:v = 4:1) was added. The mixture was vortexed and sonicated. After centrifugation at 13,000 g for 15 min at 4 °C, and the supernatant was placed at − 20 °C to precipitate proteins. After centrifugation at 13,000 g for 15 min the supernatant was ready for analysis. The LC–MS analysis was performed on a Vanquish UHPLC System (Thermo Fisher Scientific, USA) equipped with Exactive HF-X (Thermo, USA). Chromatography was carried out with an ACQUITY UPLC ®HSS T3 (2.1 × 100 mm, 1.8 µm) (Waters, Milford, MA, USA). The column was maintained at 40 °C. The flow rate and injection volume were set at 0.3 mL/min and 2 μL, respectively. For LC-ESI (+)-MS analysis, the mobile phases consisted of (B1) 0.1% formic acid in acetonitrile (v/v) and (A1) 0.1% formic acid in water (v/v). Separation was conducted under the following gradient: 0 ~ 1 min, 8% B1; 1 ~ 8 min, 8% ~ 98% B1; 8 ~ 10 min, 98% B1; 10 ~ 10.1 min, 98% ~ 8% B1; 10.1 ~ 12 min, 8% B1. For LC-ESI (-)-MS analysis, the analytes were carried out with (B2) acetonitrile and (A2) ammonium formate (5 mM) in water. Separation was conducted under the following gradient: 0 ~ 1 min, 8% B2; 1 ~ 8 min, 8% ~ 98% B2; 8 ~ 10 min, 98% B2; 10 ~ 10.1 min, 98% ~ 8% B2; 10.1 ~ 12 min, 8% B2. Metabolite identification and quantification were performed according to the protocol described previously [29]. Briefly, raw data were calculated on Compound Discoverer 3.1 (Thermo Fisher Scientific, USA) with HMDB [30] and Massbank [31] databases.

For target metabolomics, 50 μL of liquid sample (serum or cell culture supernatant) was added to 250 μL of extraction solution (methanol: acetonitrile = 1:1). Following centrifugation, evaporation and reconstitution, clear supernatant was transferred to a fresh glass vial for liquid chromatography-mass spectrometry (LC or LC–MS) analysis. LC identification was carried out using an Agilent 1260 infinity II UHPLC System (Agilent Technologies, Palo Alto, CA), equipped with a variable wavelength detector. LC separation was conducted on ZORBAX StableBond C18 (4.6 × 250 mm, 5 µm) at 40 °C using a gradient of solvent A (0.1% formic acid) and solvent B (0.1% formic acid in acetonitrile); 5 µL of sample was injected. The flow rate was 1.00 mL/min. The gradient is 0 min, 10% B; 1 min, 10% B; 8 min, 95% B; 9.5 min, 95% B; 9.6 min, 10% B; 12 min, 10% B.

LC–MS was performed using the Agilent 1260 Infinity UHPLC System (Agilent Technologies, Palo Alto, CA), equipped with the Agilent Zorbax Eclipse Plus C18 (3.5 µm, 100 mm × 2.1 mm). Mobile phases are the same as LC. The following gradient was used: 0 min, 10% B; 1 min, 10% B; 8 min, 95% B; 9.5 min, 95% B; 9.6 min, 10% B; 12 min, 10% B. The flow rate was 350 µL/min, the injection volume was 5 µL and the column temperature was 40 ºC. The standard substance was dissolved or diluted to give a final concentration of 1 µmol/L. Serial dilution was performed to prepare the calibrated standard solutions. The Agilent 6540 LC-Q-TOF–MS/MS (Agilent Technologies, Palo Alto, CA) was used to acquire mass spectrometry spectra on an information-dependent basis during the LC–MS experiment. Samples were randomized prior to LC–MS/MS analysis to minimize potential batch effects.

The metabolites of serum were analyzed by orthogonal partial least-squares discriminant analysis (OPLS-DA) and random forest in the R package ropls [32] and randomForest, respectively. The variable importance in projection (VIP) and Mean Decrease Accuracy were calculated. The pathway analysis of different metabolites was performed using MetaboAnalyst 6.0 [33] and TBtools [34].

### Enzyme activity of xanthine dehydrogenase

With the 120 nM Gallus gallus xanthine dehydrogenase (E0760c, EIAab), 50 µM indole-3-acetaldehyde (Yuanye B27461) was incubated in 1 mL of 1 M sodium pyrophosphate/0.3 mM EDTA buffer (pH = 8.5) at 37 °C for 12 h. The UV-visible absorption spectrum of the reaction product and standard samples was recorded by DS-11 + (Denovix, USA). Rapid kinetic experiments with indole-3-acetaldehyde were performed using DS-11 + with a multi-array detector. Reactions were followed at 278 nm, monitoring the increase in absorbance due to enzyme reactivity. The initial reaction mixture typically contained 4 µM enzyme and 25–250 µM substrate. For each substrate concentration used, the reaction was repeated in triplicate. Kinetic transients were fitted to a single-exponential equation, and initial rates were subsequently fitted to the Michaelis–Menten equation using Prism (v9.5.0).

### Cell cultures and reagents

IPEC-J2 cells were purchased from ATCC (Manassas, VA, USA). Cells were grown in Dulbecco’s Modified Eagle Medium (DMEM) high glucose (HyClone) supplemented with 10% fetal bovine serum (FBS; Gibco), 100 μg/mL penicillin and streptomycin in a humidified atmosphere containing 5% CO_2_ at 37 °C. Purified Indole-3-carboxaldehyde (Yuanye B27461), Indole-3-carboxylic acid (Yuanye S31552), and lipopolysaccharide (LPS; O55:B5, Biosharp, BL1386A) were dissolved in PBS. CH 223191 (Medmol, S80605) was dissolved in DMSO. All the reagents were stored at − 20 °C.

### Cell viability assessment

IPEC-J2 cells were seeded into 96-well plates at a density of 2,000 cells/well, and the IAld, ICA, or LPS were added and incubated with the cells for 24 h; then, CCK-8 (C0038, beyotime) was added into each well for another 60 min, Finally, the OD_450_ was measured by a microplate reader (Bio Tek, Winooski, VT, USA).

### Intracellular ROS assay

To determine an appropriate LPS concentration, we first calculated its IC₅₀ in IPEC-J2 cells as guided by a previous study [35]. Cells were then treated with LPS (8 µg/mL), with or without IAld and ICA, for 24 h. Then the cells were stained with the fluorescent marker 2,7-Dichlorodihydrofluorescein diacetate (DCFH-DA) (10 µM, S0033S, Beyotime). After three washes with serum-free medium, fluorescence intensity was measured on a microplate reader (BioTek, Winooski, VT, USA) at 488/525 nm (excitation/emission). Cell viability was additionally assessed with CCK-8 for normalization. Fluorescence images were captured on a Leica DMi8 fluorescence microscope using appropriate filter sets.

### In vitropermeability study

IPEC-J2 cells were seeded in 24-well Transwell® collagen-coated plates (Corning; USA), on polyester membrane filters (0.4 µm, 1.12cm^2^) [35]. Cells were cultured for up to 21 days and transepithelial electrical resistance (TEER) was measured using Millicell ERS-2 Volt-Ohm Meter (Millipore). After cells had reached confluence and formed a tight monolayer, cells were treated with PBS, LPS (8 µg/mL), LPS and IAld (50 µM), LPS and ICA (5 µM) for 24 h. After treatment, the monolayer was washed with PBS twice and 200 µL FITC-Dextran (FD-4; Maokangbio, CN) solution (1 mg/mL in HBSS) was added. After 2 h, the medium from the basal chamber was withdrawn and transferred into 96-well black opaque plates. The FD-4 concentration was determined using a microplate reader (Bio Tek, Winooski, VT, USA) at excitation and emission wavelengths of 493 and 520 nm, respectively.

### Fluorescence imaging

IPEC-J2 were plated on 24-well plates and allowed to grow overnight for NRF2 (A0674, ABclonal) and AHR (A22464, ABclonal) staining and for 3 days for ZO-1 (21,773–1-AP, Proteintech) staining. The cells were induced with vehicle (PBS), IAld (50 µM), ICA (5 µM) or LPS (8 µg/mL) for desired time points and fixed with cold paraformaldehyde. Cells were stained with primary antibodies against AHR, NRF2, or ZO-1 (1:200 dilution) overnight at 4 °C, followed by incubation with fluorescently labeled secondary antibodies (1:200 dilution; AS007, ABclonal). The nucleus was stained with DAPI (P0131, Beyotime). The fluorescence images were captured using a Leica DMi8 fluorescence microscope with appropriate laser channels.

### Real-time PCR

Total RNA was extracted from cells and tissues using TRIzol reagent (R411, Vazyme, CN). Complementary DNA was synthesized from total RNA using HiScript II Q RT SuperMix (R222, Vazyme, CN). Quantitative real-time PCR was performed using CFX Opus 384 (BIO-Rad). Gene expression values were normalized to β-actin or GAPDH and calculated using the 2^ΔΔCt^ method. Primers used in this study are listed in Additional file 1: Table S3.

### Serum diamine oxidase, LPS and inflammatory cytokines levels and intestinal mucosa SIgA levels

Serum diamine oxidase and LPS levels were measured using an Enzyme-linked immunosorbent assay (ELISA) kit (MM-60017O2 and MM-33277O2, MEIMIAN). The serum interleukin-1β (IL-1β), interferon-γ (IFN-γ), IL-6 levels were measured using the ELISA kits (MM-36910O1, MM-0520O1, MM-0521O1, MEIMIAN), respectively. Protein concentration of tissue was determined with a BCA assay kit (Beyotime, P0012). The SIgA levels of different intestinal mucosa were measured using an ELISA kit (MM-0493O1, MEIMIAN).

### Intestinal histological morphology, intestinal goblet cell numbers and immunohistochemistry analysis

Hematoxylin and eosin (H&E) staining was used to assess intestinal histological morphology. Intestinal Alcian blue (AB) staining was used to detect goblet cells. Immunohistochemistry was conducted to analyze the jejunal ZO-1 expression and distribution in chicks, following a previously described procedure [36]. The mean optical density was calculated using ImageJ software.

### Western blot

The total protein lysates were collected to prepare the samples for western blot assay, as previously described [37]. The nuclear and cytosol extraction of the IPEC-J2 cells was conducted as follows: The cells were washed with Buffer A and centrifuged at 500 g for 5 min. The pellet was lysed with Buffer A + Buffer B (2:1) and incubated for 10 min, followed by centrifugation at 12,000 g for 10 min to separate the cytoplasm (supernatant) from the nucleus and cell membrane (pellet). Buffer compositions were as follows: Buffer A consisted of 10 mmol/L HEPES (pH 7.5), 10 mmol/L KCl, 1.5 mmol/L MgCl₂, 0.5 mmol/L DTT, 1 mmol/L glycerol phosphate, and protease inhibitor cocktail (P1005, Beyotime); Buffer B was Buffer A plus 0.15% Nonidet P-40. Primary antibodies included the anti-β-actin (AC038, ABclonal), anti-ZO-1 (21,773–1-AP, Proteintech), anti-Occludin (A24601, ABclonal), anti-Claudin-1 (A2196, ABClonal), anti-Lamin B1 (12,987–1-AP, Proteintech), anti-AHR (A22464, ABclonal), and anti-NrF2 (A0674, ABclonal). Secondary antibody: HRP-conjugated anti-rabbit antibody (A24114, ABclonal).

### In silicomolecular docking analysis and Molecular dynamics (MD) simulation

The amino acid sequence and protein domain were retrieved from UniProt. The structure of xanthine dehydrogenase is obtained from AlphaFold (https://alphafold.ebi.ac.uk/entry/P47990) and the structure of xanthine dehydrogenase in complex with indole-3-acetaldehyde was determined by PyMOL (v2.5.0) using the Alignment plugin with PDB:3NVZ as a template (RMSD = 0.53). The ligand-binding domain (109–397) of AHR (*Gallus gallus*) was constructed using AlphaFold (https://alphafold.com) and validated using SAVES6.0 (https://saves.mbi.ucla.edu/). Ligand structures (IAld and ICA) were obtained from the PubChem database. The ligands were docked to the AHR protein in AutoDock Vina (v1.2.0), analyzed with PLIP (https://plip-tool.biotec.tu-dresden.de/plip-web/plip/index) and visualized in PyMOL (v2.5.0). MD simulations of the apo protein and protein–ligand complexes were performed using GROMACS 2022 [38]. The protein topologies were generated using the AMBER99SB force field. The ligand topologies were generated using the CHARMM36 force field via the CHARMM General Force Field (CGenFF) server (https://cgenff.umaryland.edu/). Each complex was solvated in a cubic box (1000 Å) using the transferable intermolecular potential with 3 points (TIP3P) water model, and neutralized with Na⁺ and Cl⁻ ions. Energy minimization was carried out for 50,000 steps using the steepest descent algorithm. During the NVT ensemble phase, the ligands were restrained, followed by further equilibration under the NPT ensemble. Equilibration for each complex was conducted for 100 ps, after which the final molecular dynamics (MD) simulation was run for 100 ns with a 2 fs time step.

### Sequence analysis and alignment

The domain of AhR protein sequences from different species is annotated by NCBI Conserved Domain Database (CDD) [39] and visualized via TBtools [34]. The potential transcriptional factor (TF) binding site of promoters was predicted by PROMO [40]. The sequence alignment of AHR LBD from different species used Multalin (http://multalin.toulouse.inra.fr/) and visualized by ESPrint [41].

### Statistical analysis

R (v4.2.2) and GraphPad Prism (v9.0) were used for statistical analysis. Growth performance was analyzed using linear mixed-effects models, with treatment as a fixed effect and cage as a random effect. Pairwise contrasts of estimated marginal means were adjusted for multiple comparisons using the Benjamini–Hochberg procedure (FDR, q < 0.05). For β-diversity and metabolomic data, permutational multivariate analysis of variance (PERMANOVA) was used to assess statistical significance. Statistical methods for all other data are described in the corresponding figure legends. A value of q < 0.05 was considered statistically significant.

## Results

### Landscape of bacteriome development and bacterial interactions during early life in chicks

**Fig. 1:**
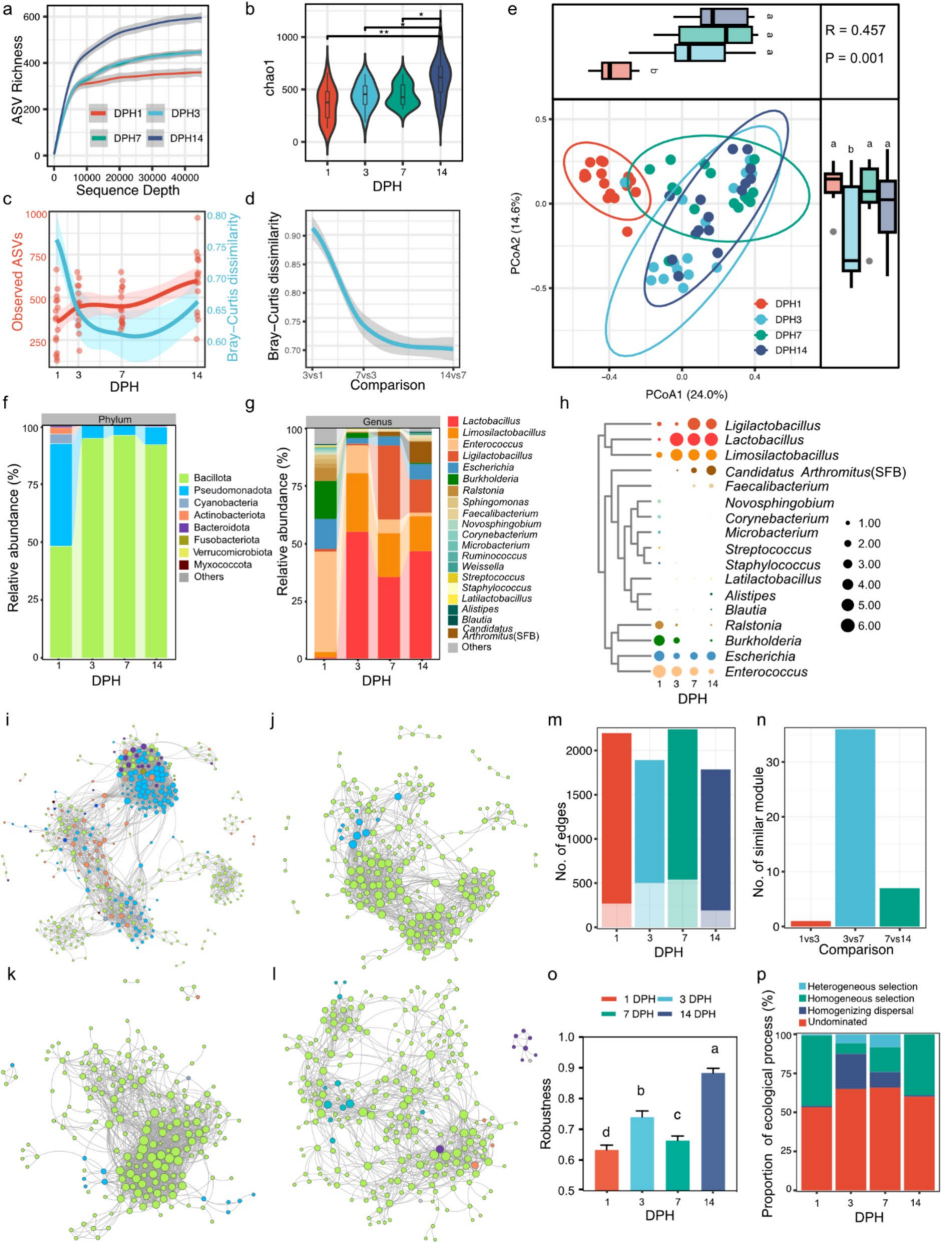
A landscape of early-life small intestine bacteriome in broiler chickens. **a** Rarefaction curves of the Amplicon Sequence Variants (ASVs) revealed by 16S rRNA gene sequencing. **b** Diversity analysis of samples from different days post hatch (DPH) on chao1 index. **c** Observed ASVs and Bray–Curtis dissimilarity of samples during the 14 DPH. **d** Pairwise comparison of Bray–Curtis dissimilarity of samples from adjacent time points. **e** PCoA of small intestinal bacterial community from different DPH based on the Bray–Curtis distance with ANOSIM analysis. **f** Bacterial community composition of the small intestine at the phylum levels. **g** Bacterial community composition of the small intestine at the genus levels. **h** Bubble heatmap showing the longitudinal occurrence patterns of the main genus (abundance > 1%) in small intestine. Abbreviation, SFB: Segmented filamentous bacteria. **i-l** The four microbial co-occurrence networks generated from the ASV in 1 DPH (**i**), 3 DPH (**j**), 7 DPH (**k**), 14 DPH (**l**). Lines represent correlations and nodes with the same color belong to the sample phylum. The colors represent the same phylum as in **f**. **m** No. of network edges over time. The deep color and light color represent positive and negative edges, respectively. **n** Pairwise comparison of similar modules from adjacent time points. **o** Robustness to hub removal of networks over time. The data are presented as the mean ± SEM and group differences were tested by one-way ANOVA. Pairwise comparisons between groups, with *p*-values adjusted by the Benjamini–Hochberg (BH) false discovery rate procedure (q < 0.05). **p** Relative importance of different ecological processes (heterogeneous selection: βNTI < − 2, homogeneous selection: βNTI > 2, dispersal limitation: |βNTI|< 2 and RCBray > 0.95, homogenizing dispersal: |βNTI|< 2 and RCBray < − 0.95, and undominated: |βNTI|< 2 and |RCBray|< 0.95) along the broiler gut bacterial community. βNTI, β-nearest taxon index; RCBray, Bray–Curtis-based Raup–Crick Index

To explore the developmental trajectory of the gut bacterial community in broiler chicks throughout the starter period (1–14 days of age), we investigated the succession of bacteria in the small intestine at 4 time points. Rarefaction curve analysis confirmed that the sequencing depth was adequate to detect nearly all bacterial species, ensuring comprehensive community profiling (Fig. 1a). Additionally, the alpha diversity tended to increase over the 1–14 day period (Fig. 1a-c). The Bray–Curtis dissimilarity between samples from the same time points indicated that the difference in the bacterial community decreased at 3 and 7 days post-hatch (DPH) but tended to increase at 14 DPH (Fig. 1c). The pairwise Bray–Curtis dissimilarity between samples from adjacent DPHs further indicated that the most substantial changes in bacterial communities occurred within the first 3 days (Fig. 1d). Principal coordinate analysis (PCoA) and nonmetric multidimensional scaling (NMDS) of beta diversity based on Bray–Curtis dissimilarity also revealed clear differences in bacterial communities among 1 DPH and 3, 7, and 14 DPH, whereas there was no significant difference between 7 and 14 DPH (Fig. 1e and Additional file 1: Fig. S1a, b). Overall, these results revealed that bacterial abundance and diversity reached a relatively stable stage at 3 DPH during the development of the foregut bacteriome.

We next explored the order and time trajectory of bacterial taxa arrival and disappearance during the starter period. Compositional analysis revealed a total of 19 phyla and 384 genera of bacteria in the small intestine, which is less than the number of bacteria in the cecum [5, 6]. At the phylum level, Bacillota and Pseudomonadota were the predominant bacterial phyla at 1 DPH, and Bacillota exhibited absolute dominance within the bacteriome after 3 DPH (Fig. 1f). At the genus level, we clustered all the bacteria into 6 clusters on the basis of their average relative abundance (Fig. 1g and Additional File 1: Fig. S1c). We visualized the increasing genera (e.g., Clusters 1 and 3) and decreasing genera (e.g., Clusters 5 and 6) to further analyze the “residents” or “stage-associated” bacteria in the foregut (Fig. 1h). For instance, the *Ligilactobacillus*, *Lactobacillus*, *Limosilactobacillus*, *Escherichia*, and *Enterococcus* genera were present during Days 1–14 and were defined as “core” bacteria. On the other hand, *Novosphingobium*, *Corynebacterium*, *Microbacterium*, *Streptococcus*, and *Staphylococcus* were observed only at 1 DPH and were assigned to “disappearance” bacteria. We also reported that *Candidatus Arthromitus* appeared at 3 DPH and increased during the starter period, which has also been reported in previous studies [42]. Notably, we observed that the relative abundance of the *Ligilactobacillus*, *Lactobacillus*, and *Limosilactobacillus* genera increased dramatically at 3 DPH and subsequently dominated the bacterial community from 3 to 14 days. These results suggest that the ability of Lactobacillaceae to colonize the small intestine of chicks is greater than that of other species and that Lactobacillaceae may play a vital role in intestinal development.

To explore the complex interactions in the chick gut, we constructed bacterial co-occurrence networks by integrating OTU-level co-occurrence analysis across the four sampling time points (Fig. 1i-l). The properties of the four networks varied considerably. We observed a greater percentage of positive edges at 14 DPH (89%) than at 3 DPH and 7 DPH (from 73 to 76%) (Fig. 1m), which suggests that collaboration is enhanced within communities and that community stability is greater at 14 DPH [43]. We compared the number of similar modules at different time points (Fig. 1n). The results revealed that 3 DPH and 7 DPH possessed the most similar modules, followed by 7 DPH and 14 DPH, which is consistent with the beta diversity results (Fig. 1c-e). Only 1 similar module was detected at 1 DPH and 3 DPH. On the basis of the random removal of module hubs, compared with the 1–7 DPH, the 14 DPH had higher robustness, and the 1 DPH had the lowest robustness (Fig. 1o).

The time-specific bacterial network prompted us to investigate the effects of the bacterial community in the small intestine. We used an ecological model to examine the internal driving forces of the time-specific community types [6]. The results indicated that deterministic processes increased from 3 to 14 DPH, whereas stochastic processes decreased during the same period. High proportions of homogeneous selection may have led to similar and stable community structures at 14 DPH (Fig. 1p).

### Lactobacillus and Ligilactobacillus were the dominant bacterial genera linked to intestine maturation

**Fig. 2:**
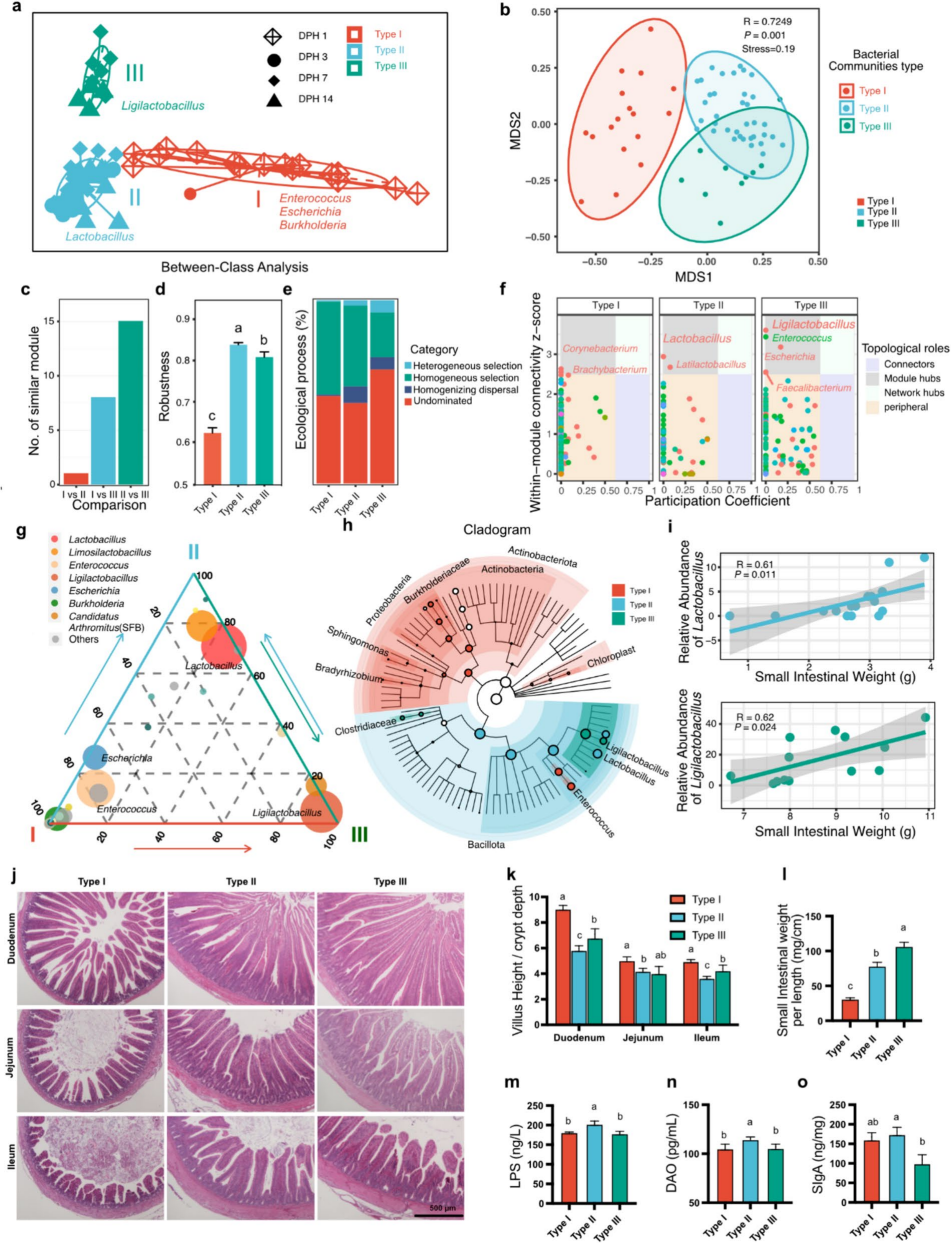
*Lactobacillus* and *Ligilactobacillus* were the dominant bacterial genera linked to intestine maturation. **a** Between-class analysis (BCA) of small intestinal bacterial communities. **b** Non-metric MultiDimensional Scaling (NMDS) of small intestinal bacterial community from different enterotypes. **c** Pairwise comparison of similar modules from different enterotypes. **d** Robustness to hub removal of networks in different enterotypes. e The relative importance of different ecological processes in different enterotypes. **f** Zi-Pi plot showing the distribution of ASVs with their topological roles in different enterotypes. Each dot represents an ASV in the bacterial network. Zi, with-module connectivity; Pi, among-module connectivity. **g** Ternary plot with enterotypes I, II, and III. The size of the dot represents the relative abundance of the genus in different enterotypes. **h** Cladogram of small intestinal bacterial composition using LEfSe. **i** The correlation between small intestinal weight and relative abundance of *Lactobacillus* and *Ligilactobacillus* in the different enterotype within the same DPH. **j** Representative images of different gut segments histological morphology by hematoxylin & eosin (HE) stained. **k** Statistical analysis of the ratio of the villus height to the crypt depth, *n* = 10. **l** Statistical analysis of small intestinal weight per length, *n* = 10. **m** Serum Lipopolysaccharide (LPS) levels, *n* = 10. **n** Serum diamine oxidase (DAO) levels, *n* = 10. **o** Intestinal mucosa SIgA levels. The data are presented as the mean ± SEM and group differences were tested by one-way ANOVA. Pairwise comparisons between groups, with *p*-values adjusted by the BH false discovery rate procedure (q < 0.05)

To further investigate bacterial communities and their relationships with intestinal development phenotypes, we first employed an unsupervised approach to classify gut bacterial communities during the starter period (1–14 days) into three distinct types. These three types also represent three distinct populations of chicks. Type I consists exclusively of 1 DPH samples, with the exception of the lowest body weight sample from 3 DPH. Type II includes samples collected between 3 and 14 DPH, characterized by a predominance of the *Lactobacillus* genus. Type III comprises samples from 7 to 14 DPH, where the *Ligilactobacillus* genus is predominant (Fig. 2a and Additional file 1: Fig. S2a). NMDS analysis of beta diversity, which was based on Bray‒Curtis dissimilarity, revealed that the three types differed significantly from each other (Fig. 2b). The bacterial community network analysis revealed that Type III had a greater proportion of positive edges than did Type II. The number of similar modules was greatest for Type III and Type II (Fig. 2c and Additional file 1: Fig. S2b). Bacterial network robustness analysis revealed that Type II and Type III bacterial communities were more robust than Type I, which indicated that Type II and Type III harbored more stable and more well-developed communities (Fig. 2d). We also observed that, compared with Type III, Type II had a higher percentage of both robust and deterministic processes, despite Type III containing a higher proportion of 14 DPH samples (40% in Type III vs. 30% in Type II, Fig. 2e).

Within-module connectivity and among-module connectivity (Zi-Pi) analyses revealed that the *Lactobacillus* and *Ligilactobacillus* genera served as key module hubs in Type II and Type III, respectively. *Latilactobacillus* and *Enterococcus* also exhibited strong within-module connectivity. These highly connected taxa are potential keystone taxa involved in foregut development (Fig. 2f).

To further confirm the dominant bacteria in different bacterial community types, we used a ternary plot, linear discriminant analysis effect size (LEfSe), and a Kruskal‒Wallis test to identify the dominant and different bacterial populations (Fig. 2g-h and Additional file 1: Fig. S2c-m). At the genus level, the dominant bacteria in Type I were *Escherichia* and *Enterococcus*. In contrast, *Lactobacillus* and *Ligilactobacillus* were the dominant genera in Types II and III, respectively. These genera also served as key module hubs in the network analysis. Further LEfSe analysis of the three bacterial communities suggested that the gut bacterial communities in Type I were enriched mainly with Pseudomonadota, including the Enterococcaceae and Burkholderiaceae families, whereas those in Type II and Type III were enriched mainly with Bacillota (Fig. 2h and Additional file 1: Fig. S2c). At the phylum level, Pseudomonadota, Cyanobacteria, Actinobacteria, and Bacteroidota were more abundant in Type I than in Type II and Type III (Additional file 1: Fig. S2d-h). At the genus level, *Lactobacillus* and *Limosilactobacillus* were more abundant in Type II, whereas *Ligilactobacillus* was more abundant in Type III (Additional file 1: Fig. S2i-k).

Probiotics are associated with gut epithelial development and can improve the intestinal epithelial barrier [2, 3]. The correlation analysis revealed a positive correlation of *Lactobacillus* and *Ligilactobacillus* with small intestinal weight (Fig. 2i). Moreover, the villus-crypt ratio and small intestinal weight were greater in Type III than in Type II in the duodenum and ileum (Fig. 2j-l). Additionally, the concentrations of LPS, DAO, and SIgA were lower in Type III than in Type II (Fig. 2 m–o). Together, *Lactobacillus* and *Ligilactobacillus* were the dominant bacterial genera in Type II and Type III, respectively, and were associated with the intestinal development and function of the intestinal epithelial barrier.

### Indole-3-carboxaldehyde is produced by L. gallinarum and L. salivarius and can be converted into indole-3-carboxylic acid

**Fig. 3:**
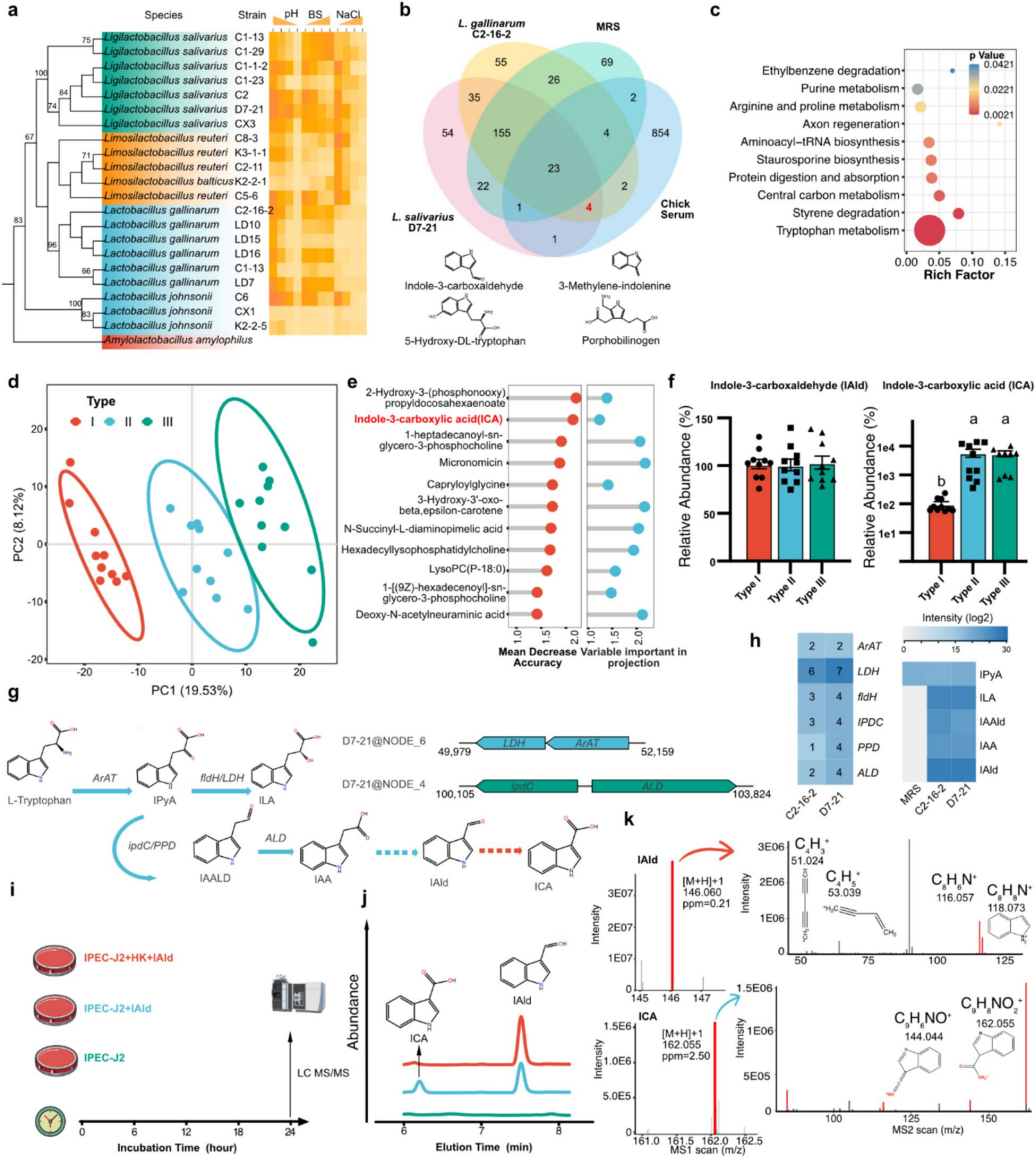
Identification of the characteristic strains and metabolites in different types of bacterial community. **a** Maximum likelihood tree of 22 family Lactobacillaceae strains based on the 16S rRNA gene with tolerance to acid, bile salts and NaCl. **b** Venn diagram analysis of metabolome in *L. gallinarum* C2-16–2, *L. salivarius* D7-21, MRS culture medium, and chick serum. The 4 common metabolites only in fermentation broth and serum are highlighed below. **c** Pathway enrichment analysis was performed using 27 common metabolites in bacterial ferment, serum and MRS culture medium by MetaboAnalyst. **d** Partial Least Squares Discriminant Analysis (PLS-DA) of serum metabolites. **e** Top 12 metabolites with the strongest influence on prediction accuracy of the random forest model and Variable importance in projection (VIP) value in the PLS-DA model. **f** Relative abundance of IAld and ICA in serum in different types of bacterial community, *n* = 10. The data are presented as the mean ± SEM and group differences were tested by one-way ANOVA. Pairwise comparisons between groups, with *p*-values adjusted by the BH false discovery rate procedure (q < 0.05) **g** Pathway schematic of bacterial and abbreviated hosts tryptophan metabolism. The representative bacterial tryptophan specific enzymes gene structure is labeled on right top. The blue arrow represents process in bacteria and the red arrow represents process in hosts. **h** The heatmap showing the number of tryptophan metabolism genes contained in different strains and the intensity of tryptophan metabolites in MRS culture medium and fermentation broth of different strains. **i** Schematic of target metabolism experiment design of IPEC-J2 cells with IAld in vitro. HK, heat-killed. **j** HPLC analysis of IAld and ICA. **k** LC–MS/MS analysis of IAld and ICA. The left panels show the MS1 spectra, representing the parent ions of IAld and ICA. The right panels display the corresponding MS2 spectra, representing the fragment ions

Guided by the genus–phenotype correlations (Fig. 2i), in which *Lactobacillus* and *Ligilactobacillus* were positively associated with small intestinal weight, we isolated 21 Lactobacillaceae strains from small intestinal chyme (including the *L. gallinarum* and *L. salivarius* clades). These isolates were benchmarked for acid tolerance, bile salt resistance, and NaCl tolerance (Fig. 3a) and for the antibacterial activity of their cell-free supernatants against *E. coli* O139 (Additional file 1: Fig. S3a). *L. salivarius* D7-21 and *L. gallinarum* C2-16–2 demonstrated the strongest tolerance and antibacterial activity among the *Ligilactobacillus* and* Lactobacillus* genera, respectively (Fig. 3a and Additional File 1: Table S2, Fig. S3a).

Bacterial metabolites are absorbed and subsequently contribute to promoting intestinal development and improving intestinal epithelial barrier function. We compared the untargeted metabolome of bacterial fermentation samples from *L. salivarius* D7-21, *L. gallinarum* C2-16–2, MRS medium, and serum, and only four metabolites (indole-3-carboxaldehyde, 3-methylene-indolenine, 5-hydroxy-DL-tryptophan, and porphobilinogen) were identified as known common metabolites after the exclusion of metabolites present in MRS medium (Fig. 3b). Three of these genes are related to tryptophan metabolism. Further metabolic pathway analysis also revealed that the top pathway term was tryptophan metabolism (Fig. 3c). Orthogonal partial least-squares discriminant analysis (OPLS-DA) revealed obvious differences in the serum metabolome among the three types of bacterial communities in chicks (Fig. 3d and Additional file 1: S3b). Moreover, we performed a random forest classifier using the abundance of metabolites as variables and combined it with the variable importance in projection (VIP) score in OPLS-DA to identify the main differentially abundant metabolites in the serum of the three types. We identified a tryptophan metabolite, indole-3-carboxylic acid (ICA), among the top 10 compounds produced through the oxidation or dehydrogenation of IAld (Fig. 3e). Interestingly, the relative abundance of IAld did not significantly differ among the serum samples in the three types of bacterial communities, whereas ICA was significantly greater in Type II and Type III than in Type I (Fig. 3f). Upon screening the bacterial metabolome database [44], we did not identify any strains capable of metabolizing and producing ICA. Therefore, we hypothesize that IAld is produced by bacteria, whereas ICA is produced by animals.

To validate our hypothesis, we performed whole-genome sequencing on *L. salivarius* D7-21 and *L. gallinarum* C2-16–2. Comparative genomics revealed that both *L. salivarius* D7-21 and *L. gallinarum* C2-16–2 are capable of producing indole derivatives. Several genes involved in tryptophan metabolism were identified in their genomes, including aminotransferase (*ArAT*), lactate dehydrogenase (*LDH*), indolelactate dehydrogenase (*fldH*), indole-3-pyruvate decarboxylase (*IPDC*), phenylpyruvate decarboxylase (*PPD*), and aldehyde dehydrogenase (*ALD*) (Fig. 3g, h and Additional File 1: Fig. S3c). *LDH* and *ArAT* are located within a single cluster, suggesting that they are regulated by the same promoter (Fig. 3g). After 4 h of monoculture, robust expression was observed, with a tryptophan-responsive increase in 3 of the 4 putative *ArAT* genes (Additional File 1: Fig. S3d-e). The metabolome also confirmed the specific tryptophan metabolites, including indole-3-lactic acid (ILA), indole-3-acetaldehyde (IAALD), indole-3-acetic acid (IAA) and IAld, in bacterial supernatants that were not detected in MRS medium (Fig. 3h). These data indicate that intact functional loci are genomically predicted in strains.

We used IPEC-J2 cells to detect IAld and its metabolites, avoiding interference from endogenous ICA detected in animal models. IPEC-J2 cells were incubated with IAld with or without heat-killed (HK) treatment prior to incubation. IPEC-J2 cells without any treatment were used as the negative control (Fig. 3i). ICA showed a prominent peak following incubation with IAld, while no detectable ICA signal was observed in either the heat-killed (HK) or negative control groups (Fig. 3j). Further LC‒MS/MS analysis also confirmed the parent and fragment ions of both IAld and ICA (Fig. 3k). Previous studies have confirmed that IAld can be catalyzed into ICA in vitro by xanthine dehydrogenase from bovine sources [45]. Here, we used purified xanthine oxidoreductase (XOR) to detect activity. The UV spectra of IAld and ICA are clearly distinct, and the reaction product of IAld with xanthine dehydrogenase exhibits a normalized spectrum identical to that of authentic ICA (Additional File 1: Fig. S3f). Under varying concentrations of IAld, the hyperbolic fit to the data yielded a V_max_ of 0.13 S^−1^ and a K_m_ of 245 µM (Additional file 1: Fig. S3g). Structural modeling revealed that IAld forms hydrogen bonds with GLU831, ARG909, and THR1039, as well as π-stacking interactions with PHE1038 and PHE943, resembling the crystal structure of xanthine dehydrogenase (Additional File 1: Fig. S3h). Together, these findings demonstrate that IAld is produced by both *L. gallinarum* C2-16–2 and *L. salivarius* D7-21 and is converted into ICA via XOR.

### L. gallinarum and L. salivarius and metabolite IAld and ICA contribute to improve intestinal epithelial barrier function and antioxidants in chicks

**Fig. 4:**
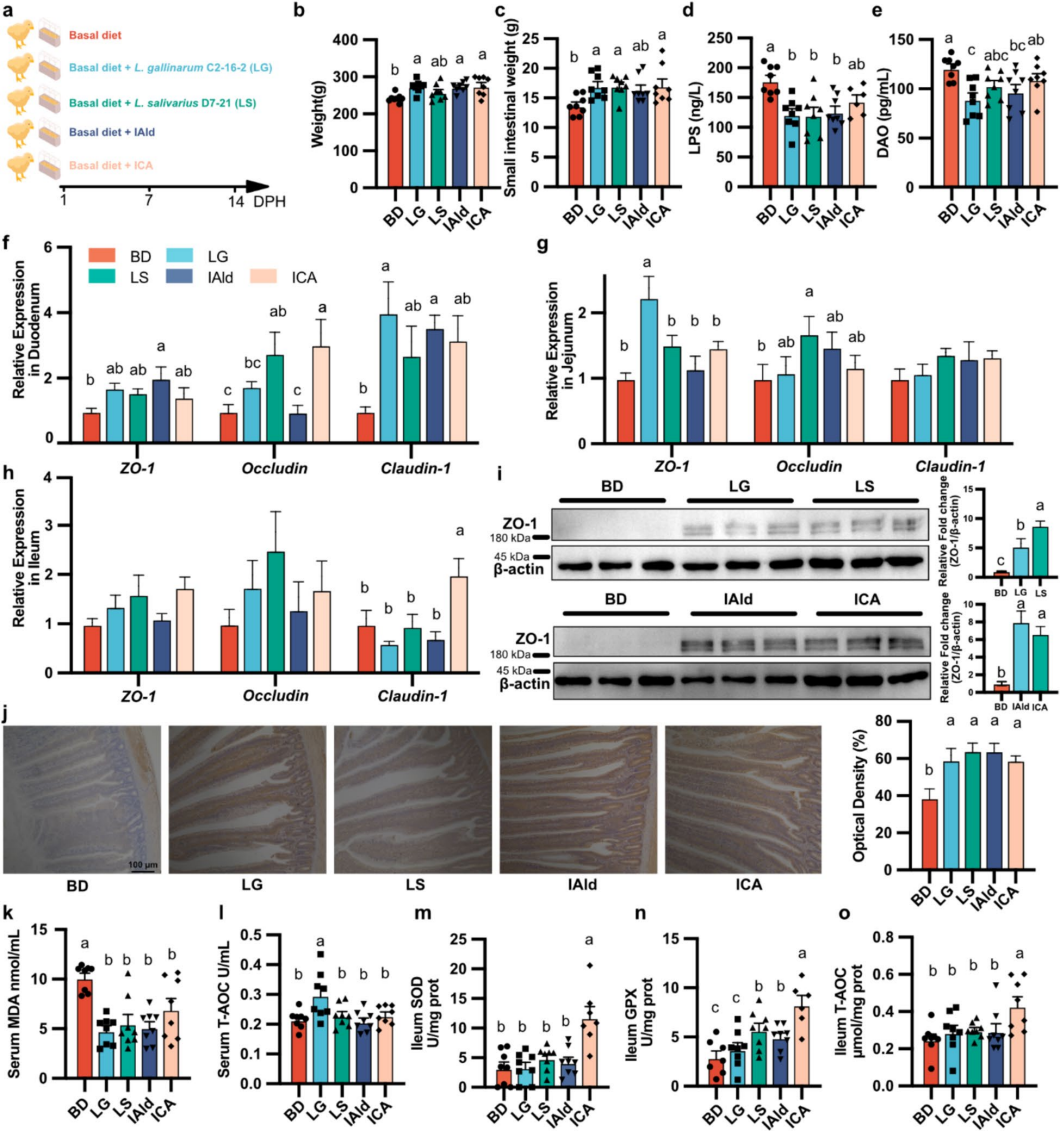
Verification of the effects of *L. gallinarum, L. salivarius* and their derived IAld and ICA on chick small intestinal development. **a** Schematic of experiment design. **b–e** Effect of LG, LS, IAld, and ICA supplementation on body weight (**b**), small intestine weight (**c**), serum LPS level (**d**), and serum DAO level (**e**) at 14 DPH, *n* = 8. **f–h** Effect of LG, LS, IAld, and ICA supplementation on expression of genes involved in tight junction protein (*ZO-1*, *Occludin*, *Claudin-1*) in duodenum (**f**), jejunum (**g**), and ileum (**h**), *n* = 8. **i** Effect of LG, LS, IAld, and ICA supplementation on expression of protein ZO-1 in duodenum. A corresponding barplot quantifies the relative gray value of ZO-1 normalized to β-actin, *n* = 3. **j** Representative images of immunohistochemical staining of the jejunal ZO-1 protein, *n* = 3. **k–m** Effect of LG, LS, IAld, and ICA supplementation on serum Malondialdehyde (MDA) (**k**), Total Antioxidant Capacity (T-AOC) (**i**), glutathione peroxidase (GPX) (**m**) levels, *n* = 8. **n–o** Effect of LG, LS, IAld, and ICA supplementation on GPX (**n**) and T-AOC (**o**) levels in the ileum. The data are presented as the mean ± SEM and group differences were tested by one-way ANOVA. Pairwise comparisons between groups, with *p*-values adjusted by the BH false discovery rate procedure (q < 0.05)

To assess the effects of *L. salivarius* D7-21, *L. gallinarum* C2-16–2, IAld, and ICA on the intestinal epithelial barrier and antioxidants in chicks, we supplemented the feed daily with *L. gallinarum* C2-16–2 (LG group), *L. salivarius* D7-21 (LS group), IAld, and ICA (Fig. 4a). The relative abundances of *L. gallinarum* and *L. salivarius* were significantly greater in the LG and LS groups, respectively, which suggested that *L. gallinarum* and *L. salivarius* successfully colonized the gut (Additional File 1: Fig. S4 a-h). Although a significant increase in serum IAld was observed only in the LG group, the serum ICA levels in the LG, LS, IAld, and ICA groups were significantly greater than those in the BD group (Additional File 1: Fig. S7a, b). These findings suggest that the IAld produced by bacterial metabolism may have been converted into ICA. We observed that the body weights of 14 DPH chicks significantly increased in the LG, IAld, and ICA groups (Fig. 4b and Additional file 1: Table S4). Additionally, compared with the BD group, the LG, LS, and ICA groups significantly increased the weight of the small intestine. Notably, the SIgA content in the jejunal mucosa increased in the LG group, whereas the SIgA levels in the ileal mucosa increased in the ICA group (Fig. 4c and Additional File 1: Fig. S4i, j; Fig. S5a, b). LG and LS significantly increased the villus-to-crypt ratio in both the duodenum and jejunum (Additional File 1: Fig. S5c-f). Moreover, the number of small intestinal goblet cells and digestive enzyme activity did not significantly differ among the five groups (Additional File 1: Fig. S6 and S7c-d).

To better evaluate the intestinal epithelial barrier, we measured serum levels of LPS and DAO, as well as the expression of tight junction proteins in the intestine, which are associated with intestinal permeability [2]. Our data indicated that supplementation with LG, LS, and IAld significantly decreased serum LPS levels and that the LG and IAld groups also presented reduced serum DAO levels (Fig. 4d, e). RT‒qPCR data revealed that LS significantly increased *Occludin* expression in the duodenum and jejunum, and LG significantly increased *ZO-1* expression in the jejunum. ICA significantly increased *Occludin* expression in the duodenum and *Claudin-1* expression in the ileum (Fig. 4f-h). The western blot and immunohistochemistry results also clearly revealed higher levels of ZO-1 protein in the LG, LS, IAld, and ICA groups than in the BD group in the duodenum and jejunum (Fig. 4i, j).

To investigate the antioxidation activity of *L. salivarius* D7-21, *L. gallinarum* C2-16–2, IAld, and ICA in vivo, we measured the redox status in serum and small intestinal segments (Fig. 4k-o and Additional file 1: Fig. S7f-t). Compared with the BD groups, the LG, LS, IAld, and ICA groups had significantly lower serum malondialdehyde (MDA) concentrations. Only the T-AOC in serum significantly increased in the LG group, and only the SOD activity and T-AOC in the ileum significantly increased in the ICA group. Both the LS and ICA groups exhibited increased GPX activity in the ileum (Fig. 4k-o and Additional file 1: Fig. S7m-o). The serum and duodenal activities of SOD and CAT did not significantly differ among the five groups. and T-AOC levels in the duodenum and jejunum also remained unchanged. Jejunal SOD activity increased significantly only in the LG group, while ileal CAT activity increased significantly only in the ICA group (Additional file 1: Fig. S7f-i, p–t). Compared with those in the BD group, the MDA levels in the treatment group did not significantly differ (Additional file 1: Fig. S7j-l). Together, these findings demonstrated that both ICA and IAld contribute to intestinal epithelial barrier function and the activity of antioxidants.

### IAld and ICA alleviated LPS-induced intestinal epithelial barrier function damage in IPEC-J2 cells

Many tryptophan derivatives have been shown to possess anti-inflammatory activity and enhance gut barrier integrity [46, 47]. To investigate the anti-inflammatory activity and intestinal epithelial integrity function of IAld and ICA, IPEC-J2 cells were treated with varying concentrations of IAld and ICA with or without LPS (Additional file 1: Fig. S8a, b). The CCK-8 results revealed that IAld at concentrations of 0–50 µM and ICA at concentrations of 0–6 µM had no significant effect on IPEC-J2 cell viability, which is consistent with the findings of previous studies using Caco2 cells and CD4 + cells [48, 49].

**Fig. 5:**
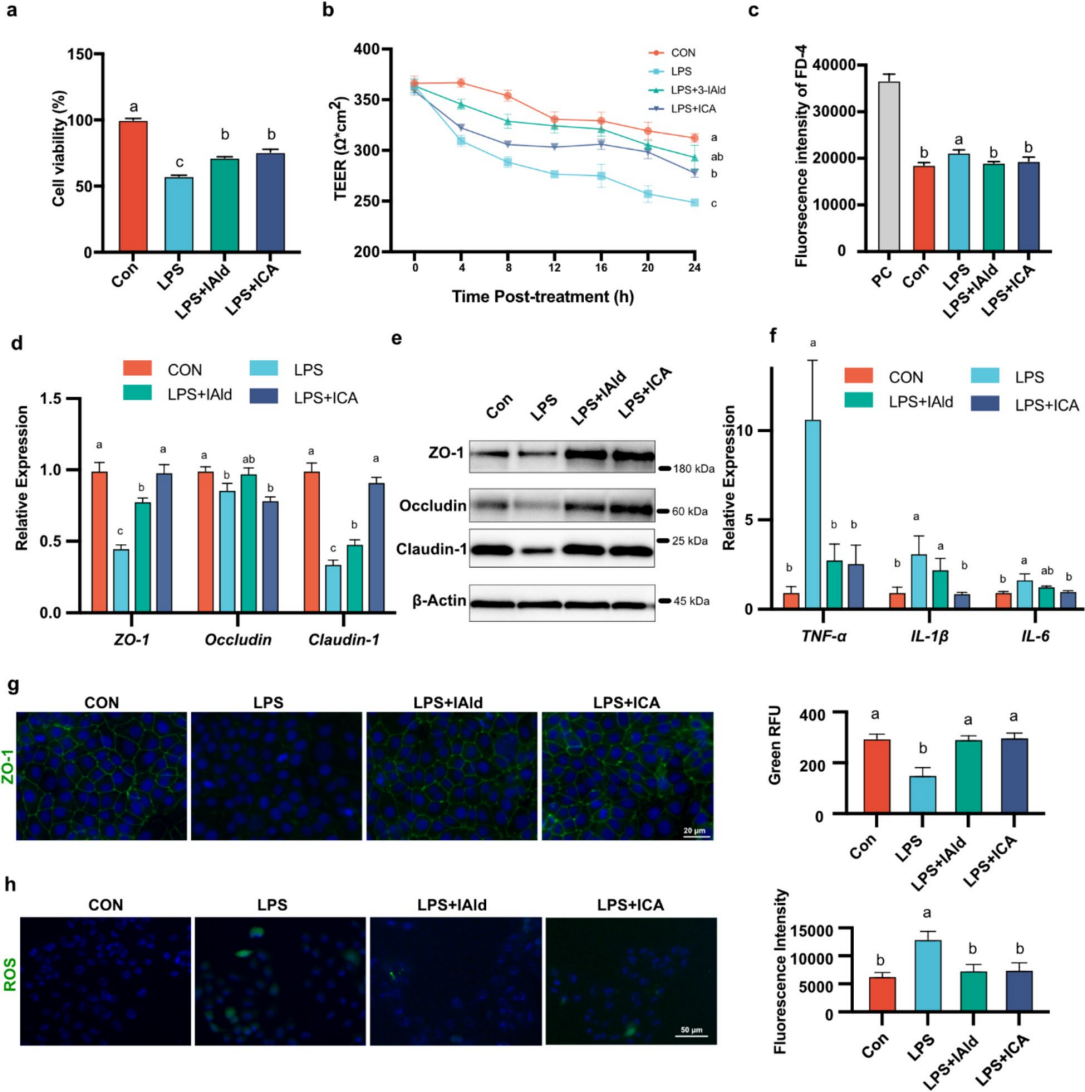
*L. gallinarum* and *L. salivarius* derived Indole-3-carboxaldehyde and metabolite Indole-3-carboxylic acid are beneficial to intestinal epithelial barrier function and reduce inflammation and oxidation in the LPS-induced model. **a** IPEC-J2 was co-treated with IAld, ICA or LPS for 24 h, and cell viability was assessed by CCK-8 assay (*n* = 3). **b** The transepithelial electrical resistance (TEER) values of the IPEC-J2 monolayers in each group were shown (*n* = 4). **c** Bar plot showing the fluorescein isothiocyanate-dextran (FD-4) intensities of the cell culture medium in the basal compartment. The positive control (PC) group represents these wells without cell seeding (*n* = 4). **d** The relative mRNA levels of Tight junction protein in each group were presented as the normalized ratio to the control (*n* = 8). **e** The representative western blot images of ZO-1, Occludin, and Claudin-1 expression in each group. **f** The relative mRNA levels of and inflammation cytokines in each group were presented as the normalized ratio to the control (*n* = 8). **g** The representative fluorescence images of ZO-1 in each group. The fluorescence intensity (*n* = 20 cell membrane regions) was measured. **h** The representative fluorescence images and fluorescence intensity of ROS in each group, *n* = 4. The data are presented as the mean ± SEM and group differences were tested by one-way ANOVA. Pairwise comparisons between groups, with *p*-values adjusted by the BH false discovery rate procedure (q < 0.05)

We used the IC50 concentration of LPS to induce tight junction damage and inflammation in the IPEC-J2 cell model (Additional File 1: Fig. S8c). Compared with LPS alone, 50 µM IAld and 6 µM ICA partially reversed the LPS-induced reductions in cell viability and barrier function, as indicated by higher CCK-8 values, higher TEER values and lower FD-4 permeability in the LPS + IAld and LPS + ICA groups (Fig. 5 a-c). We determined the mRNA and protein levels of the tight junction proteins. The relative levels of *ZO-1* and *claudin-1* in both the LPS + IAld and LPS + ICA groups were significantly greater than those in the LPS group, whereas the relative level of *occludin* did not significantly differ from that in the LPS group (Fig. 5d). Compared with LPS treatment, IAld and ICA treatment also significantly increased the protein levels of ZO-1 and occludin (Fig. 5e and Additional file 1: Fig. S8d). To investigate the anti-inflammatory activity of IAld and ICA, the expression levels of *TNF-⍺*, *IL-1β*, and *IL-6* were measured by RT‒qPCR in IPEC-J2 cells. Compared with those in the control group, the mRNA levels of *TNF-⍺*, *IL-1β*, and *IL-6* in IPEC-J2 cells significantly increased in the LPS group. Compared with the LPS group, both the LPS + IAld and LPS + ICA groups exhibited reduced *TNF-⍺* mRNA levels, while *IL-1β* and *IL-6* mRNA levels were significantly decreased only in the LPS + ICA group (Fig. 5f). Immunofluorescence analysis of the ZO-1 protein in cells revealed consistent trends in protein and mRNA levels, and treatment with IAld or ICA significantly reduced the increase in ROS levels induced by LPS (Fig. 5g, h).

### IAld and ICA enhance intestinal epithelial barrier function and antioxidant activity via AHR-NRF2 pathway

**Fig. 6:**
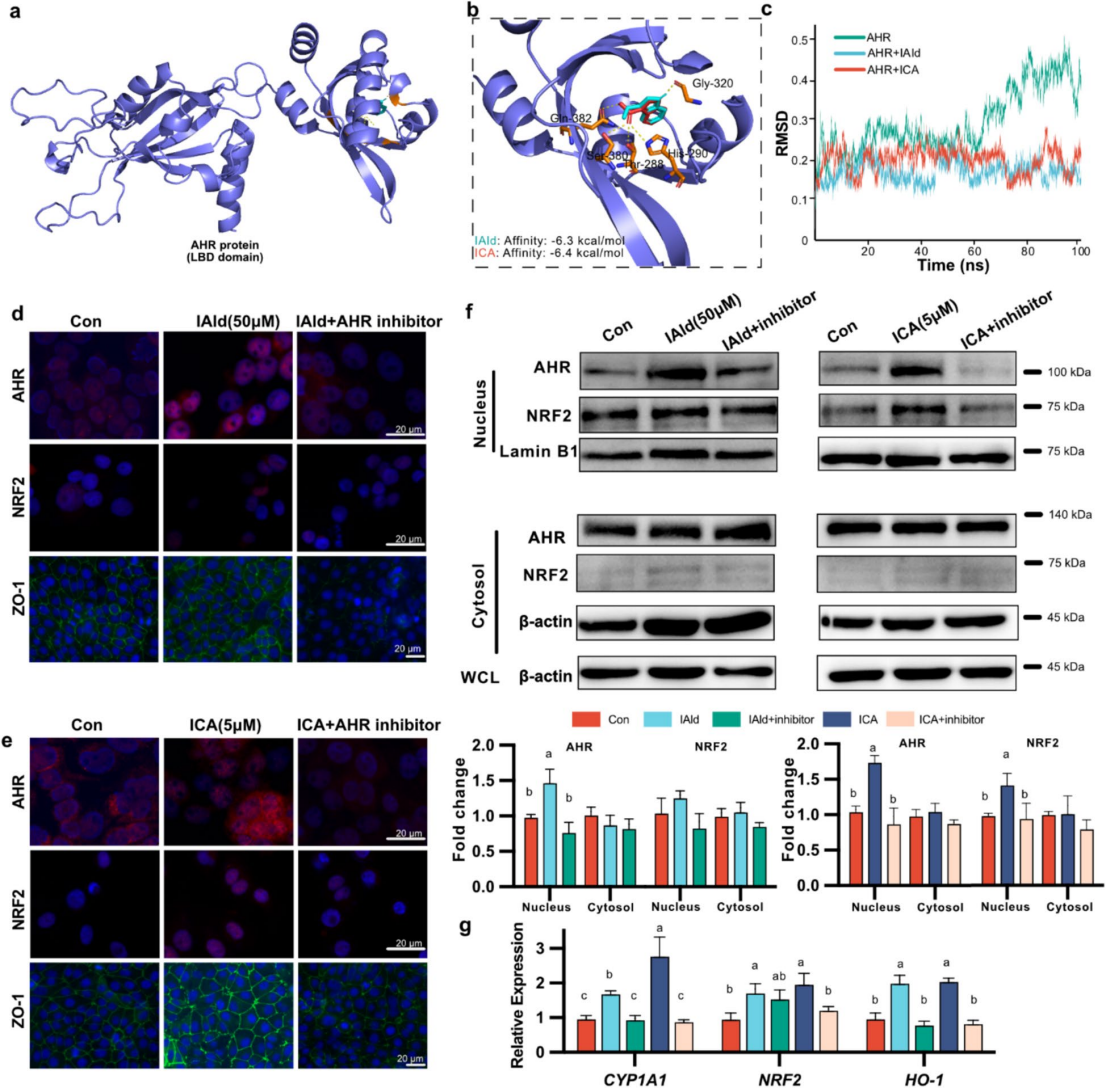
Exploration of the underlying mechanism by which IAld and ICA promote intestinal epithelial barrier function and reduce inflammation and oxidation. **a** Structure simulation of IAld and ICA with LBD of *Gallus gallus* AHR protein. **b** Molecular docking analysis of IAld and ICA on LBD of *Gallus gallus* AHR protein. IAld is shown in blue and ICA in red. Predicted molecular interactions (e.g., hydrogen bonds) are indicated by yellow dashed lines. **c** RMSD (Root mean square deviation) analysis of protein AHR LBD with or without ligand over the Molecular dynamics (MD) simulation trajectory. **d-e** Immunofluorescence images of IPEC-J2 cells treated with IAld (**d**) and ICA (**e**) with or without inhibitor. The cells were stained with anti-AHR antibody (red), ZO-1 (green), and DAPI (blue). **f** Representative western blotting of AHR and NRF2 levels in cytosol and nuclear fractions of IPEC-J2 cells. **g** IAld and ICA enhanced the relative mRNA levels of CYP1A1, NRF2, and HO-1 in IPEC-J2 cells, *n* = 4. The data are presented as the mean ± SEM and group differences were tested by one-way ANOVA. Pairwise comparisons between groups were conducted, with *p*-values adjusted by the BH false discovery rate procedure (q < 0.05)

Many tryptophan derivatives and analogs have been shown to exert their effects through the aryl hydrocarbon receptor (AHR) [10, 50, 51]. AHR is highly conserved in vertebrates and belongs to the basic helix-loop-helix/Per-ARNT-SIM (bHLH-PAS) family of transcription factors, including the bHLH domain for DNA binding, two tandemly arranged PAS domains for dimerization, and a transcription activation domain [52, 53] (Additional File 1: Fig. S9a). The ligand binding site of AHR has been mapped to its PAS-B domain, which is also referred to as the ligand binding domain (LBD) [54] (Additional File 1: Fig. S9a). Computational docking analysis of IAld and ICA with AHR revealed that both compounds can bind the LBD of AHR. IAld formed three hydrogen bonds with the LBD, whereas ICA formed four, which may explain the stronger affinity of ICA for AHR (Fig. 6a, b and Additional file 1: Table S5). Sequence alignment revealed that three of the four interacting amino acid residues are conserved among vertebrates (288THR, 320GLY, and 382GLN), whereas the 380th AA residue is a polar residue in *Gallus* (380SER) but nonpolar in humans and other mammals (Additional File 1: Fig. S9b). MD simulations also revealed that the RMSD of both the IAld-AHR complex and the ICA-AHR complex was lower than that of the AHR apo structure, which indicated that the complex structure of AHR with IAld or ICA was more stable (Fig. 6c). Surface plasmon resonance with hAHR also confirmed that ICA can bind to the LBD of AHR in vitro [48]. These results indicated that both IAld and ICA can bind to the LBD of AHR and form a relatively stable complex structure.

We examined whether AHR was activated by IAld and ICA. The results demonstrated that the translocation of AHR from the cytosol to the nucleus was promoted by both IAld and ICA treatment (Fig. 6d-f and Additional File 1: Fig. S10a). The expression of genes downstream of AHR, including *CYP1A1*, *NRF2*, and *HO-1*, significantly increased in vitro (Fig. 6g) and in vivo (Additional File 1: Fig. S10 c–e). NRF2, a downstream transcription factor (TF) of AHR, was significantly upregulated at the protein level by ICA but did not significantly change with IAld treatment (Fig. 6f). The cytokine IL-6, a downstream proinflammatory mediator, was significantly decreased in serum after ICA treatment in vivo (Additional File 1: Fig. S10f). We further observed that pretreatment with an AHR inhibitor (CH 223191) markedly attenuated the effects of both IAld and ICA (Fig. 6d-g and Additional file 1: Fig. S10b). Overall, compared with IAld, both IAld and ICA bind to AHR and activate AHR signaling, and the activity of ICA is greater even at lower concentrations.

Since AHR is required for ICA- or IAld-mediated activation, we analyzed publicly available AHR-ligand ChIP-seq data, and the results suggested that in addition to *CYP1A1*, *CYP1A2*, and *NRF2*, the tight junction proteins *ZO-1*, *occuldin*, and *claudin-1* may also be targeted by AHR (Additional File 1: Fig. S11). The protein‒protein interaction networks also predicted interactions between AHR and ZO-1 and occuldin (Additional File 1: Fig. S12 a, b). Subsequent analysis of potential TF binding sites revealed AHR or AHR:Arnt binding sites in the promoters of both *occuldin* and *ZO-1* (Additional File 1: Fig. S12c, d). These data partially elucidate the mechanisms by which IAld and ICA upregulate the expression of tight junction proteins, including ZO-1 and occludin, both in vitro and in vivo (Fig. 5f-h, Fig. 6d-e, and Additional file 1: Fig. S12).

## Discussion

Chickens, which are the most numerous and widely distributed among domesticated animals worldwide, play a vital role in global meat production [12]. In modern domestic animal production, unlike mammals, broiler chickens lack maternal contact during both the hatching process and post-hatch growth. As a result, the gut microbiota of broiler chickens is entirely derived from the environment and feed. Previous studies have quantified the gut microbiota during chicken development [5, 6]. In this study, by employing a more refined temporal sampling strategy and correlating microbial data with phenotypes, we observed significant differences in the small intestinal microbiota at very early life (3 DPH vs. 1 DPH). Furthermore, from 7 to 14 days post-hatching, the small intestinal microbiota of individual chicks predominantly stabilized into communities dominated by either *Lactobacillus* or *Ligilactobacillus*. Both are core bacterial genera in the chicken gut [55]. Here, we identified two highly active strains, *L. gallinarum* C2-16–2 and *L. salivarius* D7-21, which metabolize tryptophan into IAld, and IAld is further converted into ICA via XOR catalysis. Both metabolites activate the AHR-NRF2 pathway, enhancing tight junction integrity and reducing oxidative stress, thereby supporting intestinal health. These findings offer deeper insights into its functional role in host–microbiota interactions.

Diet, the environment, host phylogeny and evolutionary history influence the composition of the gut microbiome, contributing to host specificity [4]. Despite significant variations in the gut microbiome across different animals, some microbes have been shown to metabolize aromatic amino acids (AAAs). The microbial catabolism of AAAs results in the production of a wide range of metabolites that influence immune, metabolic, and neuronal responses locally and systemically [56]. *Clostridium sporogenes*, a human gut bacterium from the phylum Bacillus, can produce indolepropionic acid (IPA), which can fortify the intestinal barrier by directly engaging the pregnane X receptor (PXR) [57, 58]. These species also metabolize tyrosine and phenylalanine to 4-hydroxyphenylpropionic acid and 3-phenylpropionic acid (3-PPA). In pigs, *Bacteroides fragilis*-derived 3-PPA plays an important role in enhancing the intestinal epithelial barrier [2]. Recently, a study on the ruminal microbiome revealed that *Bifidobacterium pseudolongum* can convert tryptophan into IAld and accelerate the proliferation of ruminal epithelial and smooth muscle cells via the AHR-Wnt and Ca^2+^/CAMK2 signaling pathways [3]. In mice, *Bifidobacteria* with ILA-producing capacity exhibit psychobiotic potential by reducing neuroinflammation [59]. The bacterial metabolites IAld and ILA are linked to depressive symptoms in mice [60]. The physiological roles of these metabolites may vary depending on host context. Further studies are needed to understand their biological effects and assess their potential in postbiotic development.

AHR is now recognized to interact with an impressive range of chemical structures, including nonaromatic and nonhalogenated compounds, and is widely present across different animal species [61]. AHR was initially identified as a receptor strongly activated by extremely toxic chemicals, leading to the induction of its downstream target, cytochrome P4501A1. Subsequent studies revealed that endogenous molecules such as tryptophan and indole derivatives can also activate AHR, regulating a wide range of pathophysiological processes, including neuronal function, inflammatory responses, oxidative stress, immune modulation, and intestinal homeostasis [62, 63]. The sensitivity to the same metabolite varies greatly among species. In birds, *Gallus* exhibits the highest sensitivity to TCDD, whereas mallards are almost insensitive to it, and the Ser380 residue in *Gallus* AHR is likely a key amino acid contributing to this difference in sensitivity [64]. XOR is widely expressed in the intestine and liver in different animals, indicating that both the intestine and liver can metabolize IAld [65, 66]. In vitro, we observed significant cytotoxicity of ICA at a concentration of 25 µM, while the same concentration of IAld had no notable effect on IPEC-J2 cell viability (Additional File 1: Fig. S4a, b). This suggests that ICA has a relatively narrow safety concentration range. In vivo, serum ICA levels are an order of magnitude lower than those of IAld, suggesting that only a small amount of IAld is metabolized to ICA to exert its biological effects, potentially explaining XOR’s low affinity for IAld (Additional File 1: Fig. S8a, b) [48]. Notably, even a modest increase in the metabolic conversion of IAld to ICA, combined with the absorption of microbially derived IAld into the blood circulation, could significantly elevate ICA levels while allowing IAld levels to remain relatively stable (Fig. 3f). Additionally, IAld and ICA may influence chick intestinal homeostasis directly or indirectly. The increased SIgA levels in the ileal mucosa and the decreased abundance of *Escherichia* and *Enterococcus* with ICA supplementation in the feed suggest that ICA may regulate mucosal immunity and inhibit pathogens (Additional File 1: Fig. S4c, d, i). These findings emphasize the need to consider species-specific sensitivity when evaluating IAld and ICA as potential feed additives in poultry. Considering that animals may exhibit different levels of tolerance to ICA, determining optimal dosages through animal experiment remains important. Alternatively, supplementation with tryptophan, the precursor of these metabolites, could offer a safer and more universally applicable strategy.

Owing to the limitations of agricultural animal feeding practices and experimental technologies, some issues remain to be further elucidated. First, our study demonstrated that both *L. gallinarum* and *L. salivarius* were able to convert tryptophan into IAld, but the main pathway between IAA and IAld and the microbial enzyme-encoding genes involved in this process need to be further clarified. Indole-3-glyoxylic acid is likely an intermediate, but the catalytic enzyme remains unknown [50]. Second, although ICA demonstrated greater activity in vitro, its effects were not significantly superior to those of IAld in chicken experiments. This discrepancy may stem from differences in cell models or metabolite concentrations. Larger-scale studies and in vivo experiments with varying concentration gradients and different animals are needed. Moreover, while this study focused on IAld and ICA, other tryptophan metabolites, such as ILA, IAAld, and IAA, may have similar functions. However, a comparative study of the effects of these metabolites was not conducted. Further investigations are needed to elucidate the interactions among bacteria, metabolites, and animals.

## Conclusion

Overall, using 16S rRNA gene amplicon sequencing, metabolomics, comparative genomics, and animal experiments, we identified *L. gallinarum* C2-16–2 and *L. salivarius* D7-21 as two strains that contribute to small intestinal epithelial barrier function and antioxidant activity. Our findings indicated that IAld is produced by *L. gallinarum* C2-16–2 and *L. salivarius* D7-21 and can be converted into bioactive ICA. Both IAld and ICA activate AHR-NRF2 signaling to confer beneficial effects on intestinal epithelial barrier function and antioxidant capacity. These results demonstrate the complex interactions among bacteria, metabolites, and the host, highlighting the potential of targeting microbiota-derived metabolites to improve intestinal development and overall animal health.

## Supplementary Information

Additional file 1: Table S1. Basal diet composition of chicks. Table S2. Strain ranks by simple rank-aggregation. Table S3. Primers for qPCR. Table S4. Effects of different additives on growth performance of broilers. Table S5 Protein-Ligand Interaction between AHR LBD domain and IAld or ICA. Figure S1. Beta diversity and different clusters from different DPH. a NMDS of small intestinal bacterial community from different DPH based on the Bray-Curtis distance. b Pairwise comparison of Bray-Curtis distances to 1 DPH. c Bacterial time trajectory clusters over 14 DPH. Figure S2. Network analysis and comparison analysis of gut bacterial taxonomic composition. a Three microbial cooccurrence networks generated from the ASV in types I, II, and III. b No. of network edges in different types. c Bar plot showing the Linear Discriminant Analysis (LDA) scores of taxa among the three types. d-h Relative abundance of bacteria at phylum level among the three types. i-m Relative abundance of bacteria at the genus level among the three types. The data are presented as the mean ± SEM and evaluated by non-parametric Kruskal‒Wallis followed by pairwise comparisons with Benjamini–Hochberg adjustment for multiple testing. Figure S3. Screening of strains, serum metabolites and putative aromatic amino acid aminotransferase (ArAT) genes. a Bar diagram represents the diameter of the zone of inhibition on MRS culture medium. b Static result of PLS-DA in serum metabolomes analysis. The p values were calculated by 1000 permutation test. c The heatmap represents the sequence identity of the best hit of the tryptophan metabolism genes in C2-16-2 and D7-21 strains. d-e Expression level of ArAT loci in D7-21 (d) and C2-16-2 (e) of monoculture with or without 1mM tryptophan supplementation in MRS medium as measured by qRT-PCR. The expression levels are normalized to 16S rRNA gene (*n*=5). The data are presented as the mean ± SEM and evaluated by student’s t-test. Symbols indicate significance (* *, *p* < 0.01; * *p* < 0.05; ns, not significant). f UV absorption spectra of indole-3-carboxaldehyde, indole-3-carboxylic acid, and the product of the reaction of indole-3-acetaldehyde with xanthine dehydrogenase. g Plot of Kobs vs indole-3-carboxaldehyde concentration for the reaction with xanthine dehydrogenase. h Prediction of xanthine dehydrogenase complexed with indole-3-acetaladhyde. The hydrogen bonds were shown in blue dashes and the Pi-Stacking was labeled in yellow line. Figure S4. Microbial colonization, SIgA secretion, and weight of small intestine. a-h The relative abundance of specific genus or species in small intestinal contents (*n*=8). The data are presented as the mean ± SEM and evaluated by the Kruskal-Wallis test followed by pairwise comparisons with Benjamini–Hochberg (BH) adjustment for multiple comparisons. i Levels of sIgA in the duodenum, jejunum, and ileum (*n*=8). j Statistical analysis of small intestine weight (*n*=8). Statistical significance was assessed by one-way ANOVA followed by pairwise comparisons with Benjamini–Hochberg adjustment for multiple testing. Data sets marked with different letters represent a significant difference (q < 0.05). Figure S5. Analysis of intestinal histological morphology and length. a Statistical analysis of small intestinal length (*n*=8). b Statistical analysis of small intestinal weight per length (*n*=8). c-e Statistical analysis of the villus height (c), crypt depth (d), and the ratio of the villus height to the crypt depth (e). f Representative images of intestinal histological morphology by hematoxylin and eosin staining of duodenum, jejunum, and ileum, respectively (*n*=8). The data are presented as the mean ± SEM and group differences were tested by one-way ANOVA. Pairwise comparisons between groups, with p-values adjusted by the BH false discovery rate procedure (q<0.05). Figure S6. Analysis of the number of intestinal goblet cells in small intestine. a Representative images of intestinal goblet cells stained with Alcian blue (AB) staining of the duodenum, jejunum, and ileum, respectively. b Statistical analysis of goblet cell numbers in the duodenum, jejunum, and ileum, respectively (*n*=8). The data are presented as the mean ± SEM and group differences were tested by one-way ANOVA. Pairwise comparisons between groups, with p-values adjusted by the BH false discovery rate procedure (q<0.05). Figure S7. Serum and intestinal antioxidant indices and activities of digestive enzymes of intestinal contents. a-b IAld (a) and ICA (b) concentration in serum. c-e Activities of digestive enzymes in duodenal contents of chymotrypsin (c), trypsin (d), and ⍺-amylase (e). f-g Superoxide dismutase (SOD)(f) and Catalase (CAT)(g) activities in serum. h-i T-AOC levels in duodenum (h) and jejunum (i). j-l MDA levels in duodenum (j), and jejunum (k), and ileum (l). m-o GPX activities in duodenum (m), jejunum (n), ileum (o). p-q SOD activities in duodenum (p), and jejunum (q). r-t CAT activities in duodenum (r), and jejunum (s), and ileum (t). Statistical significance was assessed by one-way ANOVA followed by pairwise comparisons with Benjamini–Hochberg adjustment for multiple testing. Data sets marked with different letters represent a significant difference (q < 0.05). Figure S8. Effects of IAld and ICA on LPS-induced intestinal epithelial barrier function damage in IPEC-J2 cells. a IPEC-J2 cells were treated with IAld (0-200 µM) for 24h, (*n*=3). b IPEC-J2 cells were treated with ICA (0-200 µM) for 24h, (*n*=3). c IPEC-J2 cells were treated with LPS (0-10 µg/mL) for 24h, (*n*=3). d Quantified data of Immunoblots by Image J software. The data are presented as the mean ± SEM and group differences were tested by one-way ANOVA. Pairwise comparisons between groups, with p-values adjusted by the Benjamini–Hochberg (BH) false discovery rate procedure (q<0.05). Figure S9. AHR gene structure and sequence alignment from different animals. a Gene structure of AHR protein in different animals. b Sequence alignment of AHR Ligand Binding Domain (LBD) from different animals. Figure S10. Expression of AHR-NRF2 pathway-related genes and tight junction proteins and secretion of inflammatory cytokines. a Fluorescence intensity of AHR, NRF2, and ZO-1 in IPEC-J2 cells. The fluorescence intensity (*n* = 20 cell membrane regions) was measured. b The relative mRNA levels of Tight junction protein (ZO-1, Occludin, Claudin-1) in each group were presented as the normalized ratio to the control (*n*=4). c-e The relative mRNA levels of CYP1A1, NRF2, and HO-1 in each group were presented as the normalized ratio to the control (*n*=8). f Effects on serum inflammatory cytokine levels in each group (*n*=7). The data are presented as the mean ± SEM and group differences were tested by one-way ANOVA. Pairwise comparisons between groups, with *p*-values adjusted by the BH false discovery rate procedure (q < 0.05, # means 0.05 < q <0.06). Figure S11. AHR-ligand Chip-seq analysis. Heatmap showing publicly available ChIP-Atlas (http://chip-atlas.org/target_genes) ChIP-seq data of AHR ligand. Figure S12. Interaction of AHR and target proteins. a AHR targets NRF2 and CYP1A1 genes. b AHR also targets tight junction proteins such as TJP1 and Occludin. c AHR and NRF2 are predicted to bind promoter of occludin gene. d AHR is predicted to bind the promoter of the TJP1 gene. 

## Data Availability

The accessions for the 16S rRNA gene sequencing data and whole-genome sequences in this study are available in the National Center for Biotechnology Information (NCBI) under accessions: PRJNA1225209 and PRJNA1314736.

## Abbreviations

ALD: Aldehyde dehydrogenase
ArAT: Aromatic amino acid aminotransferase
ASVs: Amplicon sequence variants
CYP1A1: Cytochrome P450, family 1, subfamily A
DAO: Diamine oxidase
DPH: Day post-hatching
fldH: Indolelactate dehydrogenase
IAA: Indole-3-acetic acid
IAALD: Indole-3-acetaldehyde
IAld: Indole-3-carboxaldehyde
IC50: Half maximal inhibitory concentration
ICA: Indole-3-carboxylic acid
IL-6: Interleukin 6
ILA: Indole-3-Lactic acid
INF-γ: Interferon gamma
IPDC: Indole-3-pyruvate decarboxylase
LDH: Lactate dehydrogenase
LEfSe: Linear discriminant analysis effect size
LPS: Lipopolysaccharide
MDA: Malondialdehyde
MRS: De Man, Rogosa and Sharpe
NMDS: Nonmetric multidimensional scaling
OPLS-DA: Orthogonal partial least-squares discriminant analysis
PcoA: Principal coordinates analysis
PPD: Phenylpyruvate decarboxylase
RMSD: Root mean square deviation
ROS: Reactive oxygen species
SIgA: Secretory Immunoglobulin A
TJP: Tight junction protein
TNF-α: Tumor necrosis factor alpha
VIP: Important in projection
ZO-1: Zonula Occludens-1
