# Supplementary material for: Gut microbiome-derived indole-3-carboxaldehyde promotes intestinal development via AHR-NRF2 signaling in the early-life of chicks
Source: Microbiome. 2025 Dec 16;14:34. doi: 10.1186/s40168-025-02289-2 (PMC12821288; doi:10.1186/s40168-025-02289-2)
Supplement: Supplementary file 2 — Additional file 1: Table S1. Basal diet composition of chicks. Table S2. Strain ranks by simple rank-aggregation. Table S3. Primers for qPCR. Table S4. Effects of different additives on growth performance of broilers. Table S5 Protein-Ligand Interaction between AHR LBD domain and IAld or ICA. Figure S1. Beta diversity and different clusters from different DPH. a NMDS of small intestinal bacterial community from different DPH based on the Bray-Curtis distance. b Pairwise comparison of Bray-Curtis distances to 1 DPH. c Bacterial time trajectory clusters over 14 DPH. Figure S2. Network analysis and comparison analysis of gut bacterial taxonomic composition. a Three microbial cooccurrence networks generated from the ASV in types I, II, and III. b No. of network edges in different types. c Bar plot showing the Linear Discriminant Analysis (LDA) scores of taxa among the three types. d-h Relative abundance of bacteria at phylum level among the three types. i-m Relative abundance of bacteria at the genus level among the three types. The data are presented as the mean ± SEM and evaluated by non-parametric Kruskal‒Wallis followed by pairwise comparisons with Benjamini–Hochberg adjustment for multiple testing. Figure S3. Screening of strains, serum metabolites and putative aromatic amino acid aminotransferase (ArAT) genes. a Bar diagram represents the diameter of the zone of inhibition on MRS culture medium. b Static result of PLS-DA in serum metabolomes analysis. The p values were calculated by 1000 permutation test. c The heatmap represents the sequence identity of the best hit of the tryptophan metabolism genes in C2-16-2 and D7-21 strains. d-e Expression level of ArAT loci in D7-21 (d) and C2-16-2 (e) of monoculture with or without 1mM tryptophan supplementation in MRS medium as measured by qRT-PCR. The expression levels are normalized to 16S rRNA gene (n=5). The data are presented as the mean ± SEM and evaluated by student’s t-test. Symbols indicate significance (* *, p < [file 40168_2025_2289_MOESM1_ESM.pdf]

Table S1. Basal diet composition of chicks.

Items	Chick diet
Ingredient (%)	
Corn	59.44
Soybean meal	33.07
Limestone	2.00
Soybean oil	2.00
$CaHPO_4$	2.10
Premix[a]	0.30
Choline chloride	0.15
NaCl	0.3
Lys	0.24
Thr	0.20
Met	0.20
Total	100
Calculated composition[b] (%)	
ME (MJ/kg)	11.92
CP	20.84
Ca	2.03
Available phosphorus	0.44
Lys	1.28
Methionine	0.51
Met+Cys	0.85

[a]The premix provided the following per kg of diet: Vitamin A 10,280 IU, Vitamin D 2,280 IU, Vitamin E 17.12 mg, Vitamin K 6.82 mg, Vitamin B1 2.28mg, Vitamin B2 5.68 mg, Vitamin B6 2.28 mg, niacin 22.84 mg, folic acid 1.12 mg, biotin 0.18 mg, Fe 100 mg, Cu 8 mg, I 0.9 mg, Mn 20 mg, and Zn100 mg.
[b]Values were calculated from data provide by the China Feed Database (2020).

Table S2. Strain ranks by simple rank-aggregation.

Strain	Rank _pH3	Rank _BS4	Rank NaCl	Rank inhibition	Rank Borda sum	Rank Borda
Ligilactobacillus salivarius D7-21	1	9	1	2	13	1
Ligilactobacillus salivarius C1-29	13	1	3	6	23	2
Lactobacillus gallinarum C2-16-2	7	5	7	5	24	3
Ligilactobacillus salivarius C2	2	15	4	3	24	3
Lactobacillus gallinarum LD7	8	3	6	15	32	5
Ligilactobacillus salivarius CX3	6	6	8	15	35	6
Ligilactobacillus salivarius C1-13	15	2	11	9	37	7
Lactobacillus johnsonii C6	3	12	19	4	38	8
Limosilactobacillus reuteri C5-6	5	13	20	1	39	9
Lactobacillus gallinarum LD16	9	4	12	15	40	10
Lactobacillus gallinarum LD10	11	7	14	15	47	11
Limosilactobacillus reuteri K3-1-1	14	17	9	9	49	12
Ligilactobacillus salivarius C1-1-2	4	10	21	15	50	13
Limosilactobacillus reuteri C8-3	18	11	13	9	51	14
Limosilactobacillus reuteri C2-11	19	19	5	8	51	14
Ligilactobacillus salivarius C1-13	12	14	16	9	51	14
Limosilactobacillus balticus K2-2-1	21	8	15	9	53	17
Lactobacillus johnsonii K2-2-5	15	20	10	9	54	18
Ligilactobacillus salivarius C1-23	20	18	2	15	55	19
Lactobacillus gallinarum LD15	10	16	16	15	57	20

Table S3. Primers for qPCR.

Genes	Species	Orientation	Sequences (5′-3′)	Accession Number
pig-GAPDH	*Sus scrofa*	Forward	GATGGTGAAGGTCGGAGTGAAC	NM_001206359.1
		Reversed	TGGGTGGAATCATACTGGAACA	
pig-Occludin	*Sus scrofa*	Forward	ACGAGCAGCAAAGGATTCTTC	NM_001163647.2
		Reversed	TCACACCCAGGATAGCACTCATT	
pig-Claudin-1	*Sus scrofa*	Forward	TGCCTCAGTGGAAGATTTACTCC	NM_001244539.1
		Reversed	TGGTGTTCAGATTCAGCAAGGA	
pig_ZO-1	*Sus scrofa*	Forward	AGTTTGATAGTGGCGTTGACAC	XM_005659811.1
		Reversed	GCTGAAGGACTCACAGGAACA	
pig_Actin	*Sus scrofa*	Forward	PGATGAGATTGGCATGGCTTT	AY550069.1
		Reversed	CACCTTCACCGTTCCAGTTT	
Pig-TNF-α	*Sus scrofa*	Forward	TGCCTACTGCACTTCGAGGTTATC	NM_214022
		Reversed	CAGATAAGCCCGTCGCCCAC	
pig_IL-1β	*Sus scrofa*	Forward	AATTCGAGTCTGCCCTGTACCC	NM_001005149
		Reversed	GCCAAGATATAACCGACTTCACCA	
pig_IL-6	*Sus scrofa*	Forward	CAGAGATTTTGCCGAGGATG	NM_214399
		Reversed	TGGCTACTGCCTTCCCTACC	
pig_CYP1A1	*Sus scrofa*	Forward	CAGAGCTGCTTAGCCTTATCAACC	NM_214412.1
		Reversed	CTGGATGCTGGGATTTGTCACCAG	
pig_NRF2	*Sus scrofa*	Forward	AACCAAACCGACAGAAATTGACAAC	XM_013984303.2
		Reversed	TGGAGAGGATGCTGCTGAAGG	
pig_HO-1	*Sus scrofa*	Forward	AGGCTGAGAATGCCGAGTTC	NM_001004027.1
		Reversed	TGTGGTACAAGGACGCCATC	
chick_ACTIN	*Gallus gallus*	Forward	AGTACCCCATTGAACACGGT	X00182.1
		Reversed	ATACATGGCTGGGGTGTTGA	
chick_CYP1A1	*Gallus gallus*	Forward	GGACTCATTGATTGGGCACT	NM_205147.2
		Reversed	CGTACATCATGCACCAGGAC	
chick_NRF2	*Gallus gallus*	Forward	ACGCTTTCTTCAGGGGTAGC	NM_205117.2
		Reversed	GTTCGGTGCAGAAGAGGTGA	
chick_HO-1	*Gallus gallus*	Forward	GTCGTTGGCAAGAAGCATCC	NM_205344.2
		Reversed	GGGCCTTTTGGGCGATTTTC	
chick_ZO-1	*Gallus gallus*	Forward	CCACTGCCTACACCACCATCTC	XM_040680624.2
		Reversed	CGTGTCACTGGGGTCCTTCAT	
chick_CLDN1	*Gallus gallus*	Forward	GCATGGAGGATGACCAGGTGA	NM_001013611.2
		Reversed	GAGCCACTCTGTTGCCATACCAT	
chick_OCLN	*Gallus gallus*	Forward	GTCTGTGGGTTCCTCATC	NM_205128
		Reversed	CCAGTAGATGTTGGCTTTG	

16S rRNA	*Lactobacillus gallinarum*	Forward	TAAGCCGTTACCTTACCA	EF468093.1
		Reversed	TAATGACGCTGGGGAC	
16S rRNA	*Ligilactobacillus salivarius*	Forward	TACCACGGATGCTTGCATT	OK037470.1
		Reversed	AGGATCATGCGATCCTTAGAGA	
uidA	*Escherichia coli*	Forward	GTCCAAAGCGGCGATTTG	S69414
		Reversed	CAGGCCAGAAGTTCTTTTTCCA	
16S rRNA	*Enterococcus faecalis*	Forward	ATCAGAGGGGGATAACACTT	OK271984.1
		Reversed	ACTCTCATCCTTGTTCTTCTC	
16S rRNA	*Limosilactobacillus reuteri*	Forward	GCCGCCTAAGGTGGGACAGAT	MZ208825.1
		Reversed	AACACTCAAGGATTGTCTGA	
16S-23S rRNA	*Lactobacillus johnsonii*	Forward	AGAGAGAAACTCAACTTGAAATA	OR183795.1
		Reversed	CCTTCATTAACCTTAACAGTTAA	
16S-23S rRNA	*Lactobacillus plantarum*	Forward	ATTCATAGTCTAGTTGGAGGT	OR183794.1
		Reversed	CCTGAACTGAGAGAATTTGA	
16S rRNA	*Lactobacillus spp*	Forward	TGGAAACAGRTGCTAATACCG	This study
		Reversed	GTCCATTGTGGAAGATTCCC	
16S rRNA(891-1033)	N/A	Forward	TGGAGCATGTGGTTTAATTCGA	This study
		Reversed	TGCGGGACTTAACCCAACA	
LDH	*Ligilactobacillus salivarius*	Forward	GTCGTGGCCCAGTTGTAGAT	This study
		Reversed	TAGAAGCCAACATGCGGTGT	
D7-21_ARAT_1	*Ligilactobacillus salivarius*	Forward	AAGATGCAGCTCAAGAAGCCT	This study
		Reversed	AATGCACCTTGAGGTTTGGC	
D7-21_ARAT_2	*Lactobacillus gallinarum*	Forward	ACGGGCAAACACGTTTCTTTA	This study
		Reversed	ATTCTCCAGCCTGTCATCGC	
LG-ARAT_1	*Lactobacillus gallinarum*	Forward	ACTTCTTGACGCCATCAGCA	This study
		Reversed	GCCCATACAGGAGTAGGCAC	
LG-ARAT_2	*Lactobacillus gallinarum*	Forward	GGATGCAAGCAGTAAAGCCG	This study
		Reversed	ACATAACCTTCACCGCCAGG	

Table S4. Effects of different additives on growth performance of broilers.

Index	BD	LG	LS	IAld	ICA
Initial body weight, g	42.0±0.4	41.9±0.5	41.8±0.5	41.8±0.5	41.9±0.4
7 days body weight, g	113.3±5.7^{b}	129.1±4.3^{a}	122.6±4.3^{a}	121.9±8.1^{ab}	123.0±5.4^{a}
14 days body weight, g	242.4±10.2^{b}	274.0±15.3^{a}	258.3±20.1^{ab}	272.8±12.9^{a}	275.7±23.9^{a}
7 days body weight gain, g	71.3±5.8^{b}	87.2±4.0^{a}	80.8±4.2^{a}	79.7±8.1^{ab}	81.2±5.2^{a}
14 days body weight gain, g	200.3±10.1^{b}	232.0±14.9^{a}	216.5±20.2^{ab}	231.0±12.9^{a}	233.9±23.9^{a}
7 days Feed intake, g	95.5±8.2^{a}	112.2±5.8^{b}	106.9±4.0^{ab}	103.6±13.1^{ab}	107.7±9.0^{ab}
14 days Feed intake, g	389.1±11.2^{b}	421.8±8.4^{a}	405.2±15.8^{ab}	390.7±30.3^{b}	407.6±13.1^{ab}
7 days Gain/Feed, g/g	0.750±0.074	0.779±0.056	0.755±0.033	0.773±0.055	0.755±0.036
14 days Gain/Feed, g/g	0.515±0.031^{b}	0.550±0.040^{ab}	0.535±0.047^{ab}	0.595±0.058^{a}	0.574±0.055^{ab}

Data are means ± standard deviation (n = 8) and labeled means without a common letter differ, q<0.05. BD, base diet; LG, BD supplemented with *Lactobacillus gallinarum* C2-16-2 (10^{11} CFU/kg); LS, BD supplemented with *Ligilactobacillus salivarius* D7-21(10^{11} CFU/kg); IAld, BD supplemented with Indole-3-carboxaldehyde (0.1g/kg); ICA, BD supplemented with indole-3-carboxylic acid (0.1g/kg).

Table S5 Protein–Ligand Interaction between AHR LBD domain and IAld or ICA

Index	Residue	AA	Distance	Ligand Atom	Interaction	Ligand
1	288	THR	3.31	2314	Hydrophobic	IAld
2	294	PHE	3.92	2310	Hydrophobic	IAld
3	296	PRO	3.97	2313	Hydrophobic	IAld
4	296	PRO	3.88	2315	Hydrophobic	IAld
5	307	LEU	3.79	2314	Hydrophobic	IAld
6	314	LEU	3.49	2315	Hydrophobic	IAld
7	323	PHE	3.41	2313	Hydrophobic	IAld
8	352	LEU	3.56	2310	Hydrophobic	IAld
9	320	GLY	1.79, 2.75	2307(Npl)	Hydrogen	IAld
10	380	SER	2.20, 3.07	2318(O2)	Hydrogen	IAld
11	382	GLN	2.80, 3.00	2318(O2)	Hydrogen	IAld
12	290	HIS	4.88	2307,2308,2309,2310,2311	π-Stacking	IAld
13	290	HIS	4.83	2308,2309,2312,2313,2314,2315	π-Stacking	IAld
14	288	THR	3.19	2312	Hydrophobic	ICA
15	294	PHE	3.99	2310	Hydrophobic	ICA
16	296	PRO	3.99	2315	Hydrophobic	ICA
17	307	LEU	3.68	2314	Hydrophobic	ICA
18	314	LEU	3.14	2315	Hydrophobic	ICA
19	323	PHE	3.65	2313	Hydrophobic	ICA
20	352	LEU	3.81	2310	Hydrophobic	ICA
21	320	GLY	1.90, 2.85	2307(Npl)	Hydrogen	ICA
22	380	SER	2.51, 3.23	2318(O.co2)	Hydrogen	ICA
23	382	GLN	2.80, 3.19	2319(O.co2)	Hydrogen	ICA
24	382	GLN	2.23, 2.96	2319(O.co2)	Hydrogen	ICA
25	290	HIS	4.78	2307,2308,2309,2310,2311	π-Stacking	ICA
26	323	PHE	4.84	2308,2309,2312,2313,2314,2315	π-Stacking	ICA
27	290	HIS	4.79	2318,2319	Salt Bridge	ICA

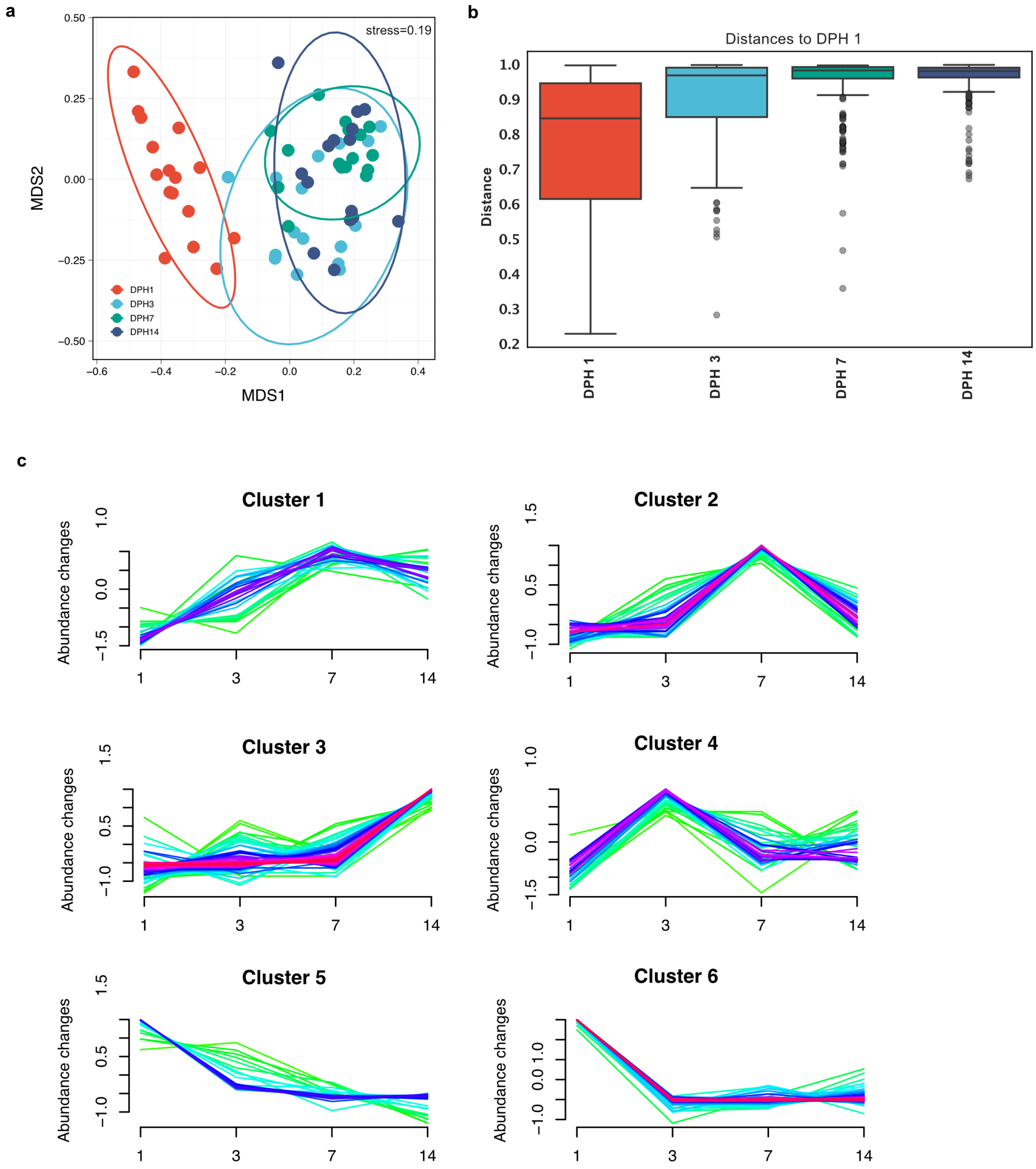

Figure S1. Beta diversity and different clusters from different DPH.

a NMDS of small intestinal bacterial community from different DPH based on the Bray–Curtis distance.

b Pairwise comparison of Bray–Curtis distances to 1 DPH.

c Bacterial time trajectory clusters over 14 DPH.

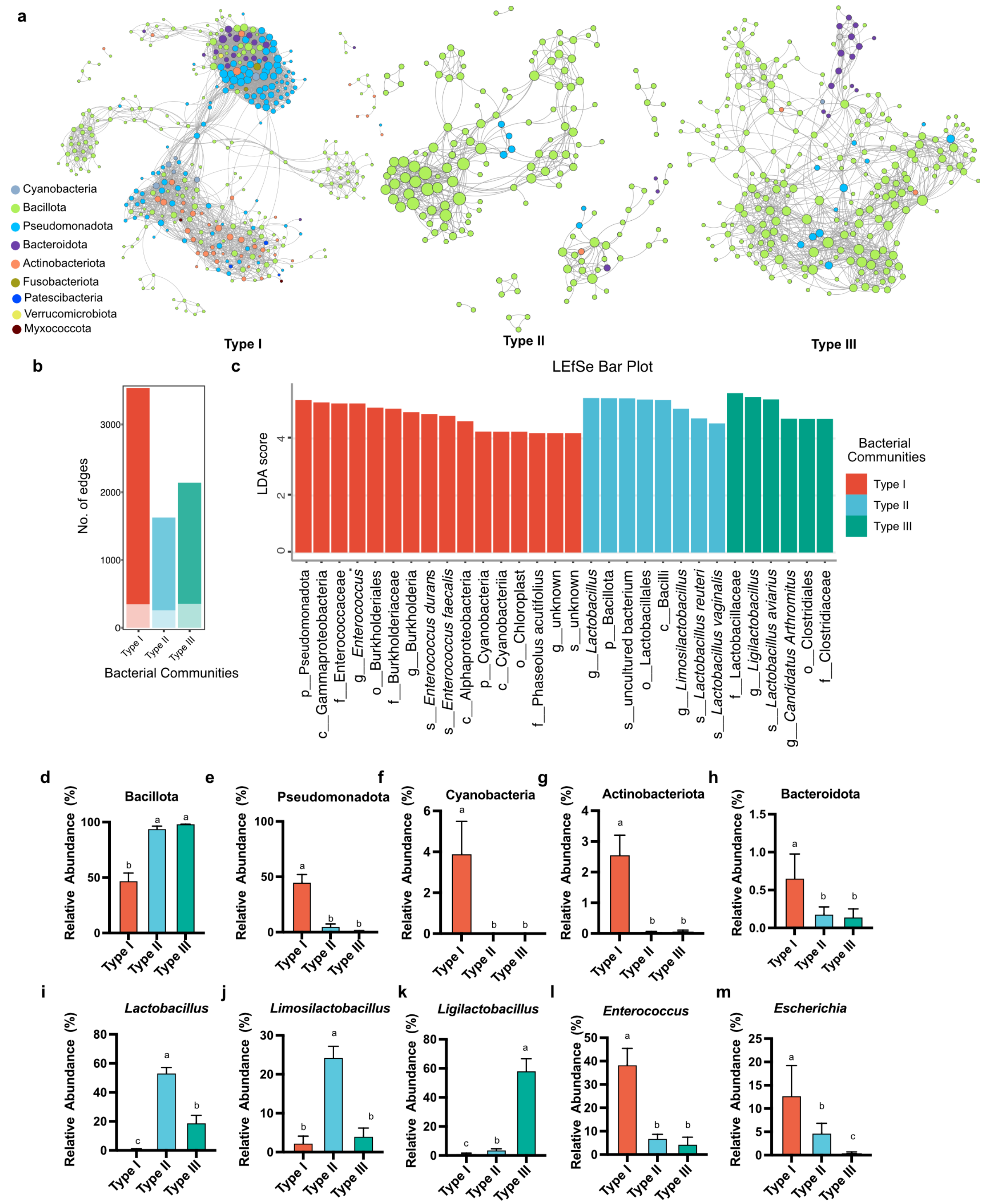

Figure S2. Network analysis and comparison analysis of gut bacterial taxonomic composition.
a Three microbial cooccurrence networks generated from the ASV in types I, II, and III.
b No. of network edges in different types.
c Bar plot showing the Linear Discriminant Analysis (LDA) scores of taxa among the three types.
d-h Relative abundance of bacteria at phylum level among the three types.
i-m Relative abundance of bacteria at the genus level among the three types. The data are presented as the mean ± SEM and evaluated by non-parametric Kruskal–Wallis followed by Dunn's multiple comparisons.

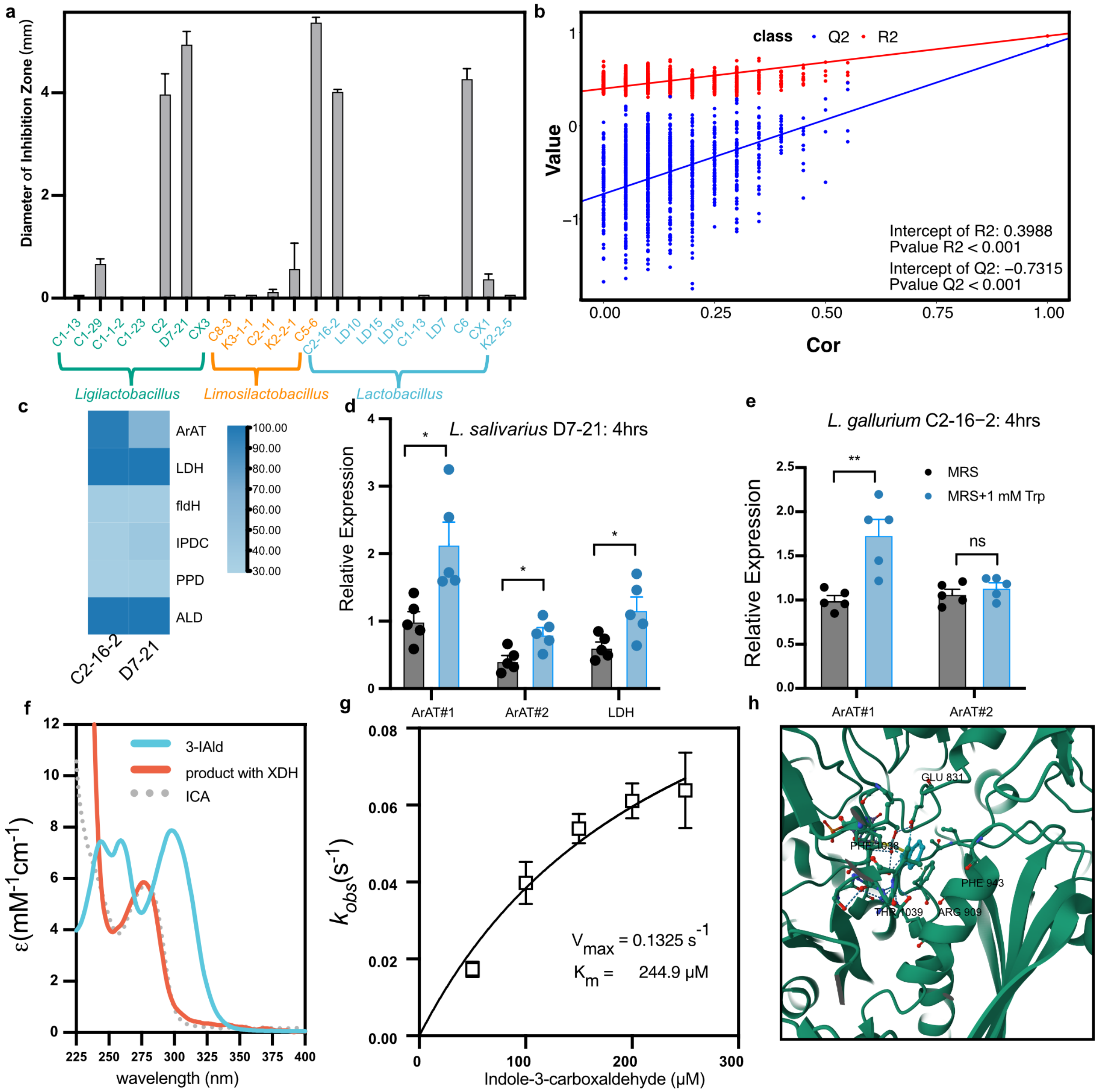

Figure S3. Screening of strains, serum metabolites and putative aromatic amino acid aminotransferase (*ArAT*) genes.

a Bar diagram represents the diameter of the zone of inhibition on MRS culture medium.

b Static result of PLS-DA in serum metabolomes analysis. The *p* values were calculated by 1000 permutation test.

c The heatmap represents the sequence identity of the best hit of the tryptophan metabolism genes in C2-16–2 and D7-21 strains.

d-e Expression level of *ArAT* loci in D7-21 (**d**) and C2-16-2 (**e**) of monoculture with or without 1mM tryptophan supplementation in MRS medium as measured by qRT-PCR. The expression levels are normalized to 16S rRNA gene (n=5). The data are presented as the mean ± SEM and evaluated by student's t-test. Symbols indicate significance (* *, $p < 0.01$; * $p < 0.05$; ns, not significant)

f UV absorption spectra of indole-3-acetaladhyde, indole-3-carboxylic acid, and the product of the reaction of indole-3-acetaldehyde with xanthine dehydrogenase.

g Plot of K_{obs} vs Indole-3-carboxaldehyde concentration for the reaction with xanthine dehydrogenase.

h Prediction of xanthine dehydrogenase complexed with indole-3-acetaladhyde. The hydrogen bonds were shown in blue dashes and the Pi-Stacking was labeled in yellow line.

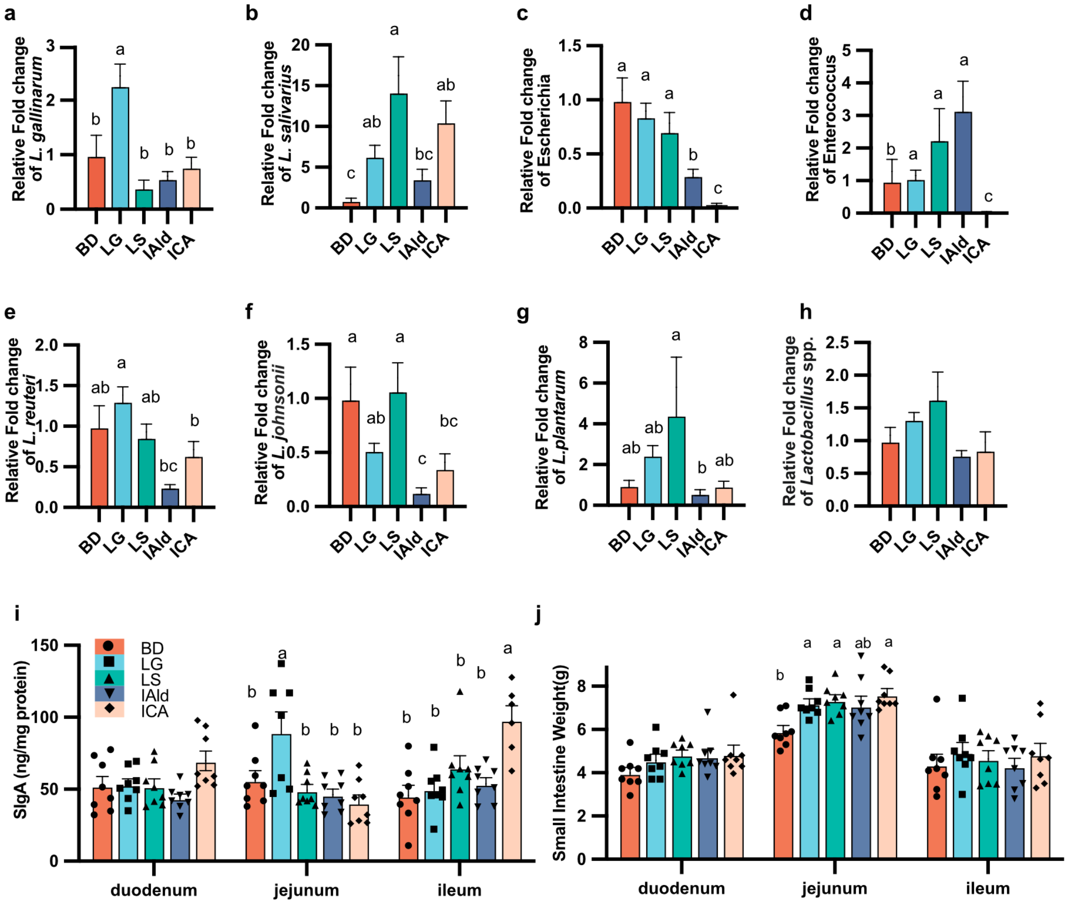

Figure S4. Microbial colonization, SIgA secretion, and weight of small intestine

a-h The relative abundance of specific genus or species in small intestinal contents (n = 8). The data are presented as the mean ± SEM and evaluated by the Kruskal–Wallis test followed by pairwise comparisons with Benjamini–Hochberg (BH) adjustment for multiple comparisons

i Levels of sIgA in the duodenum, jejunum, and ileum (n = 8).

j Statistical analysis of small intestine weight (n = 8). Statistical significance was assessed by one-way ANOVA followed by pairwise comparisons with Benjamini–Hochberg adjustment for multiple testing. Data sets marked different letters represent a significant difference ($q < 0.05$).

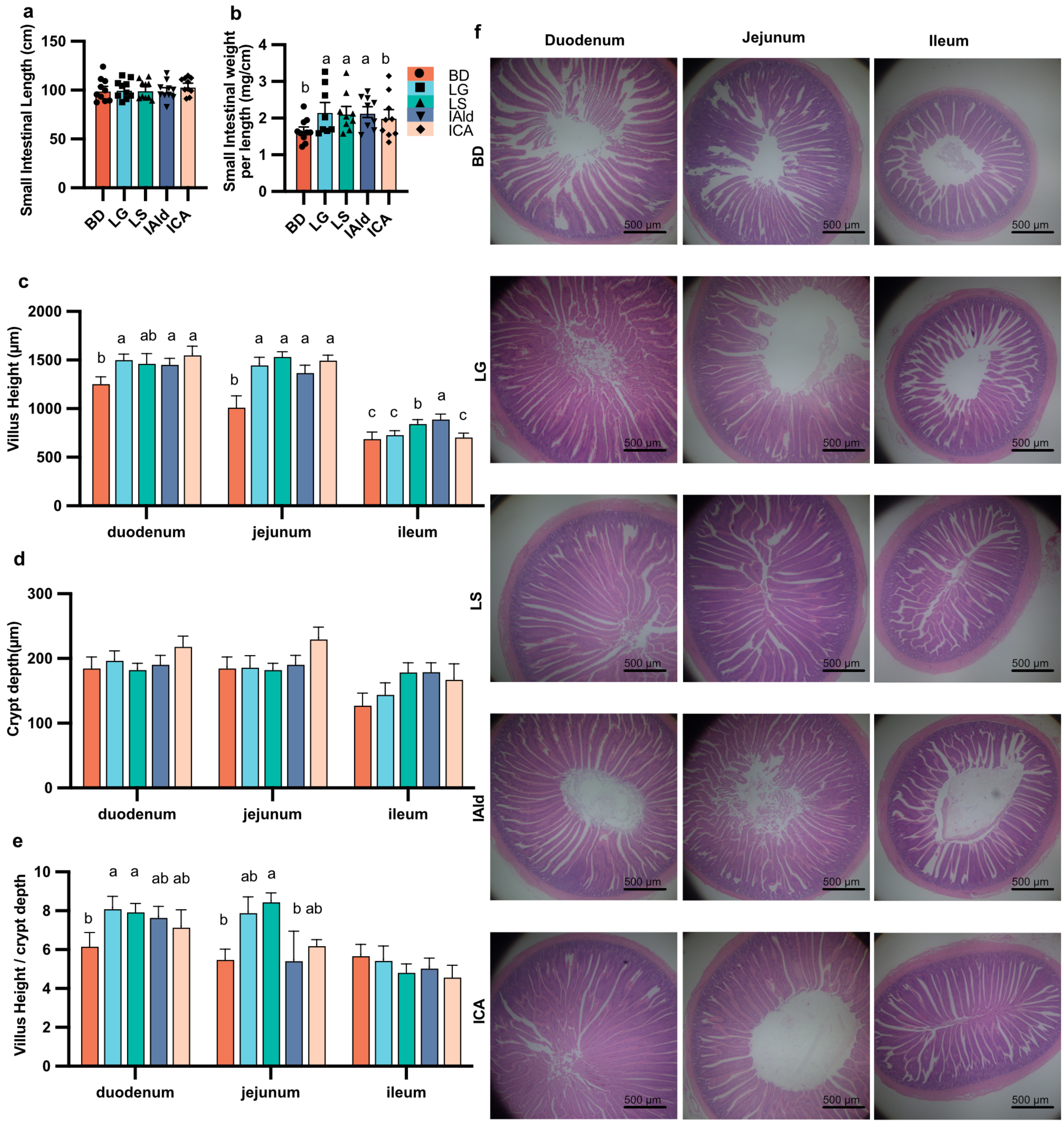

Figure S5. Analysis of intestinal histological morphology and length.
a Statistical analysis of small intestinal length (n = 8).
b Statistical analysis of small intestinal weight per length (n = 8).
c-e Statistical analysis of the villus height (**c**), crypt depth (**d**), and the ratio of the villus height to the crypt depth (**e**).
f Representative images of intestinal histological morphology by hematoxylin and eosin staining of duodenum, jejunum, and ileum, respectively. (n = 8). Statistical significance was assessed by one-way ANOVA followed by pairwise comparisons with Benjamini–Hochberg adjustment for multiple testing. Data sets marked different letters represent a significant difference (q < 0.05).

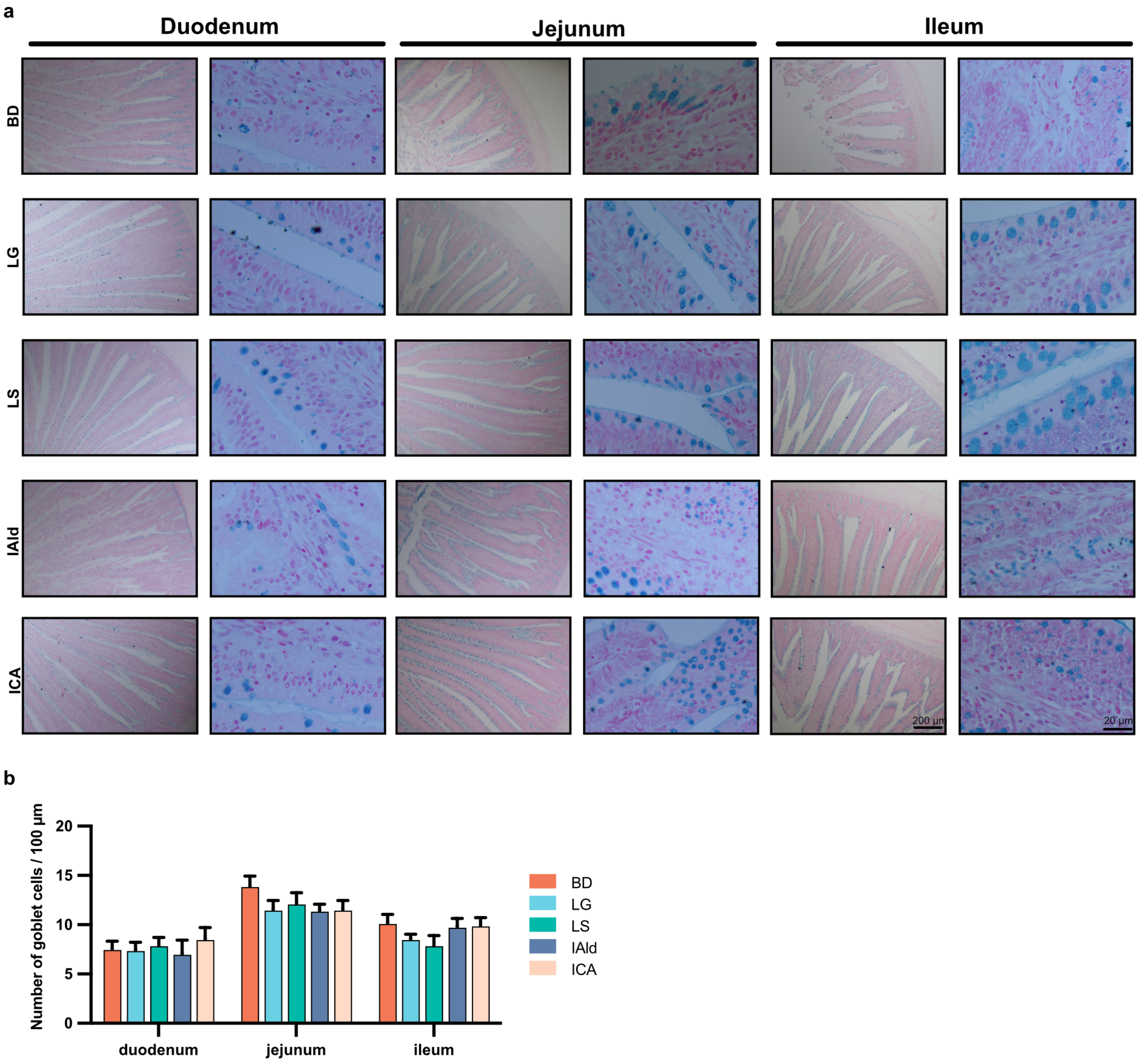

Figure S6. Analysis of the number of intestinal goblet cells in small intestine.

a Representative images of intestinal goblet cells stained with Alcian blue (AB) staining of duodenum, jejunum, and ileum, respectively.

b Statistical analysis of goblet cell numbers in the duodenum, jejunum, and ileum, respectively (n = 8). Statistical significance was assessed by one-way ANOVA followed by pairwise comparisons with Benjamini–Hochberg adjustment for multiple testing.

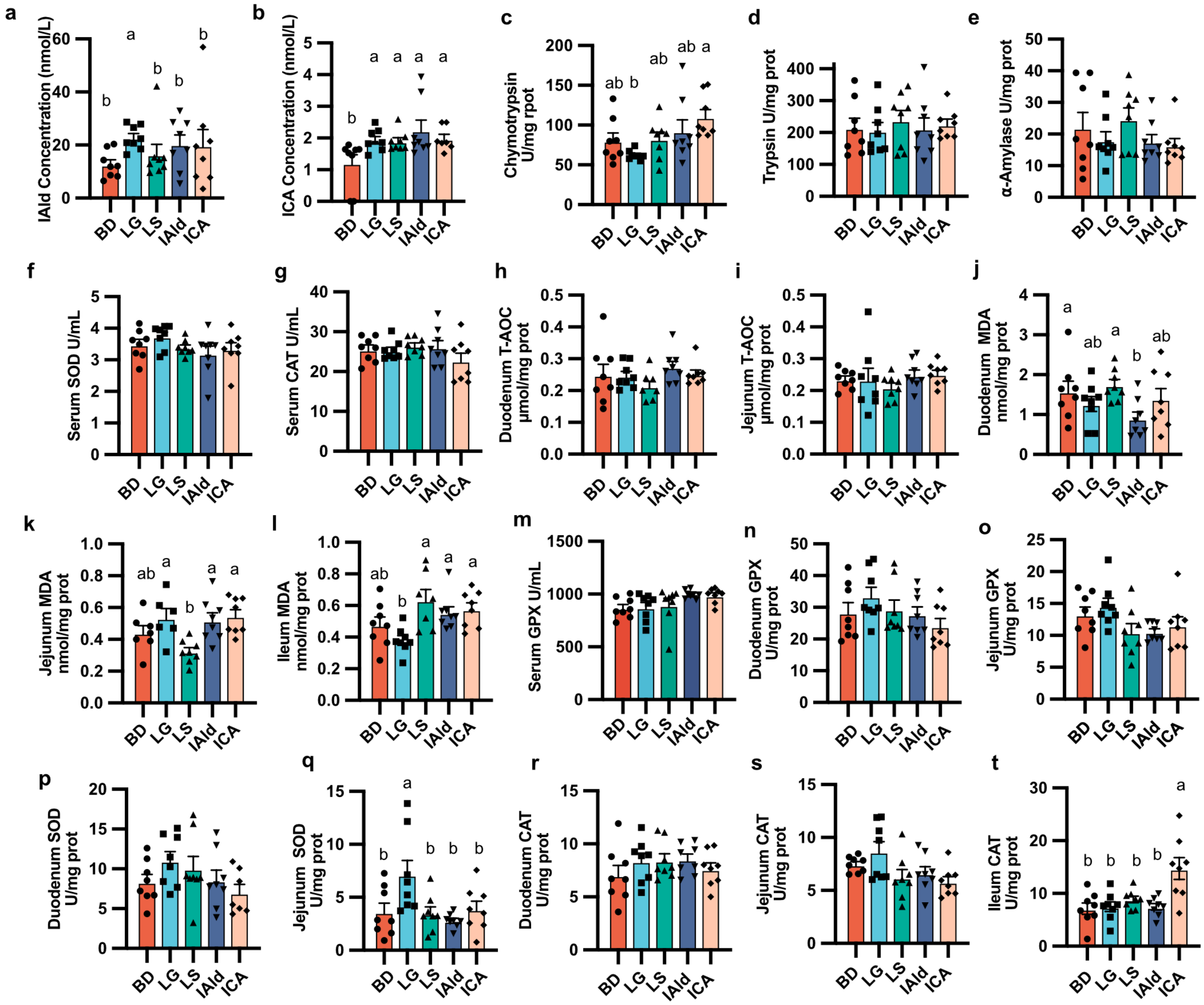

Figure S7. Serum and intestinal antioxidant indices and activities of digestive enzymes of intestinal contents.

a-b IAld (**a**) and ICA (**b**) concentration in serum.

c-e Activities of digestive enzymes in duodenal contents of chymotrypsin (**c**), trypsin (**d**), and α-amylase (**e**).

f-g Superoxide dismutase (SOD)(**f**) and Catalase (CAT)(**g**) activities in serum.

h-i T-AOC levels in duodenum (**h**) and jejunum (**i**).

j-l MDA levels in duodenum (**j**), and jejunum (**k**), and ileum (**l**).

m-o GPX activities in duodenum (**m**), and jejunum (**n**), ileum (**o**).

p-q SOD activities in duodenum (**p**), and jejunum (**q**).

r-t CAT activities in duodenum (**r**), and jejunum (**s**), and ileum (**t**). Statistical significance was assessed by one-way ANOVA followed by pairwise comparisons with Benjamini–Hochberg adjustment for multiple testing. Data sets marked different letters represent a significant difference ($q < 0.05$).

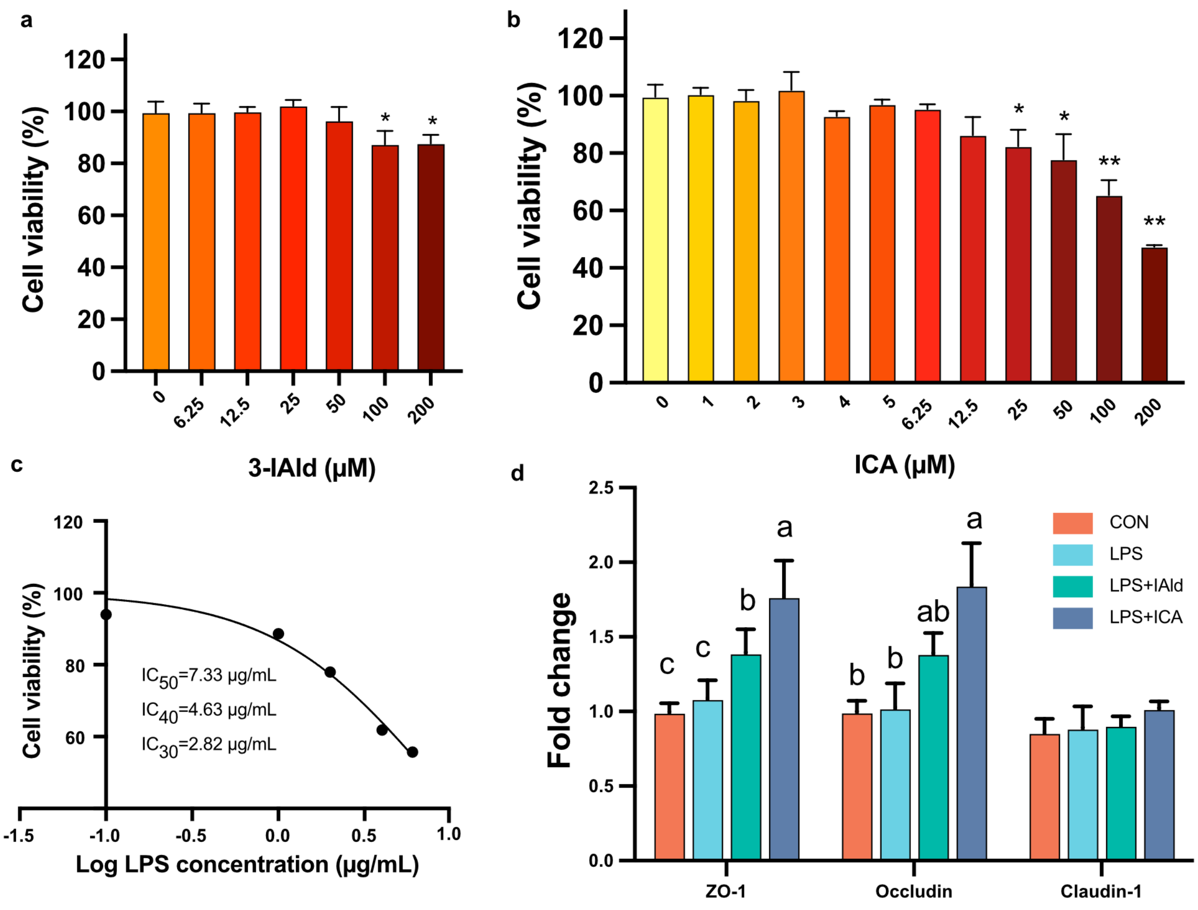

Figure S8. Effects of IAld and ICA on LPS induced intestinal epithelial barrier function damage in IPEC-J2 cells.

a IPEC-J2 cells were treated with IAld (0-200 μM) for 24 h (n = 3).

b IPEC-J2 cells were treated with ICA (0-200 μM) for 24 h (n = 3). * $p < 0.05$, ** $p < 0.01$ compared with 0 group.

c IPEC-J2 cells were treated with LPS (0-10 μg/mL) for 24 h (n = 3).

d Quantified data of Immunoblots by Image J software. Statistical significance was assessed by one-way ANOVA followed by pairwise comparisons with Benjamini–Hochberg adjustment for multiple testing. Data sets marked different letters represent a significant difference ($q < 0.05$).

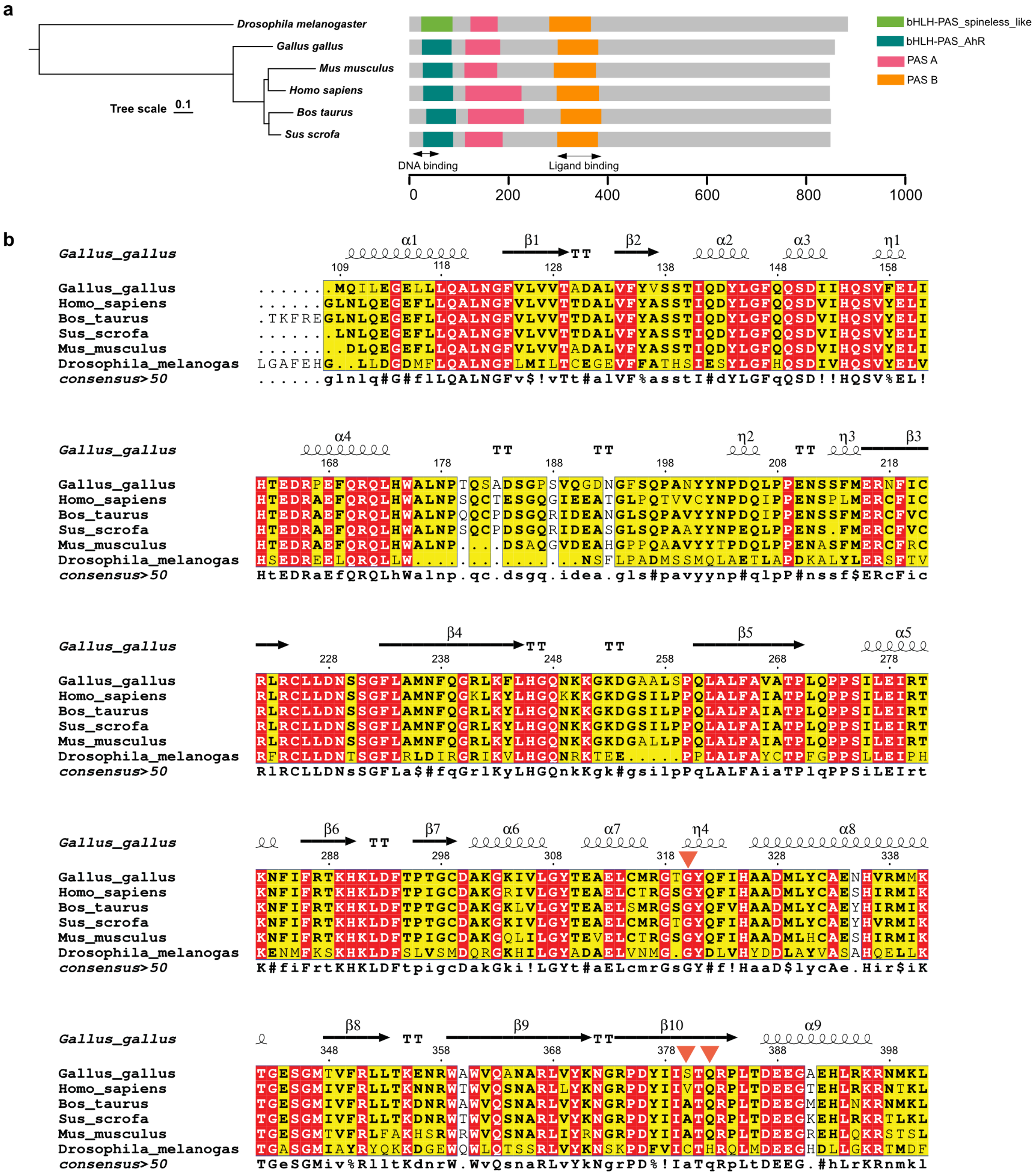

Figure S9. AHR gene structure and sequence alignment from different animals.
a Gene structure of AHR protein in different animals.
b Sequence alignment of AHR Ligand Binding Domain (LBD) from different animals.

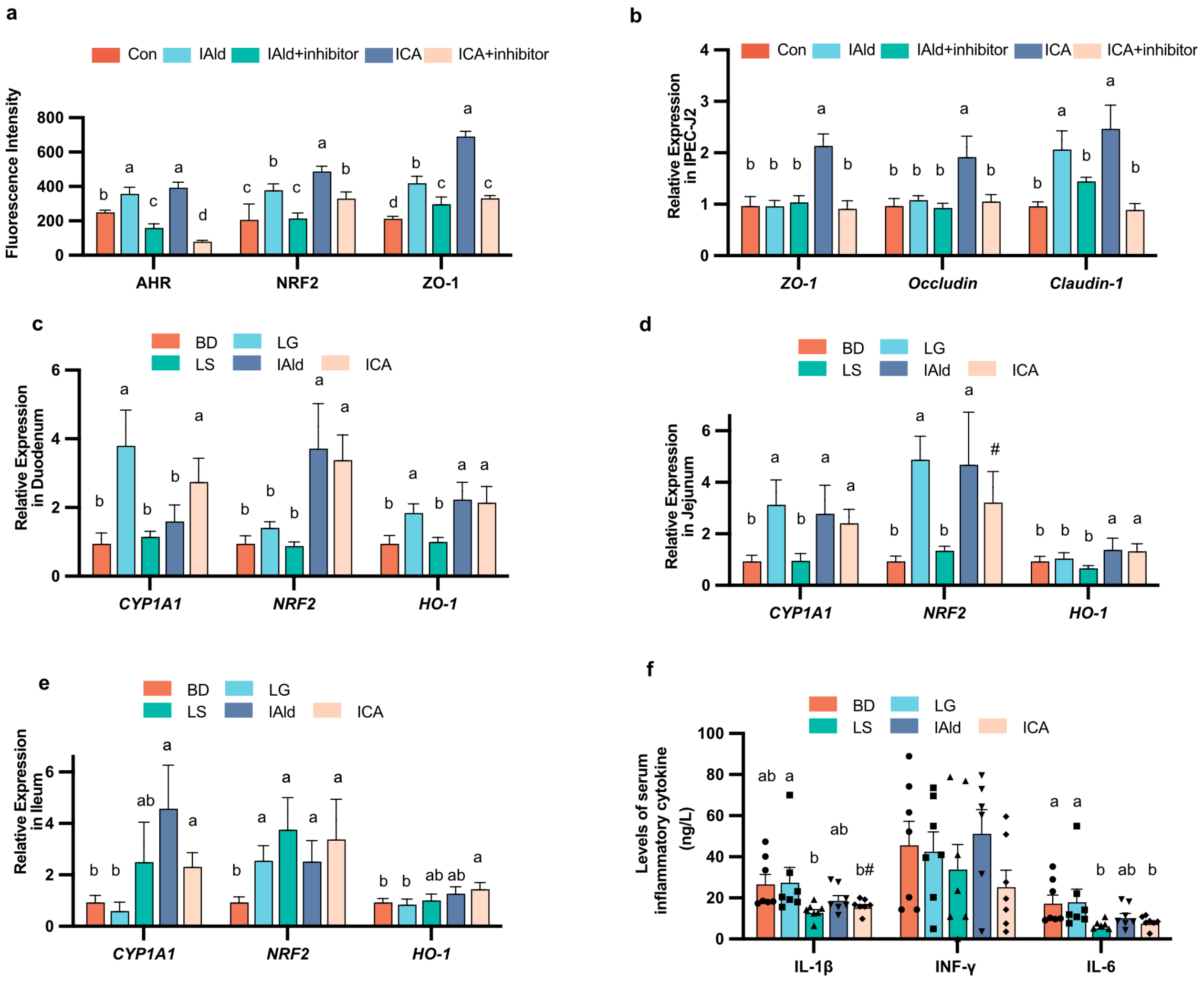

Figure S10. Expression of AHR-NRF2 pathway-related genes and tight junction proteins and secretion of inflammatory cytokines.

a Fluorescence intensity of AHR, NRF2, and ZO-1 in IPEC-J2 cells. The fluorescence intensity (n = 20 cell membrane regions) was measured.

b The relative mRNA levels of Tight junction protein (*ZO-1*, *Occludin*, *Claudin-1*) in each group were presented as the normalized ratio to the control (n=4).

c-e The relative mRNA levels of *CYP1A1*, *NRF2*, and *HO-1* in each group were presented as the normalized ratio to the control (n=8).

f Effects on serum inflammatory cytokine levels in each group (n=7). Statistical significance was assessed by one-way ANOVA followed by pairwise comparisons with Benjamini–Hochberg adjustment for multiple testing. Data sets marked different letters represent a significant difference ($p < 0.05$, # means $0.05 < p < 0.06$).

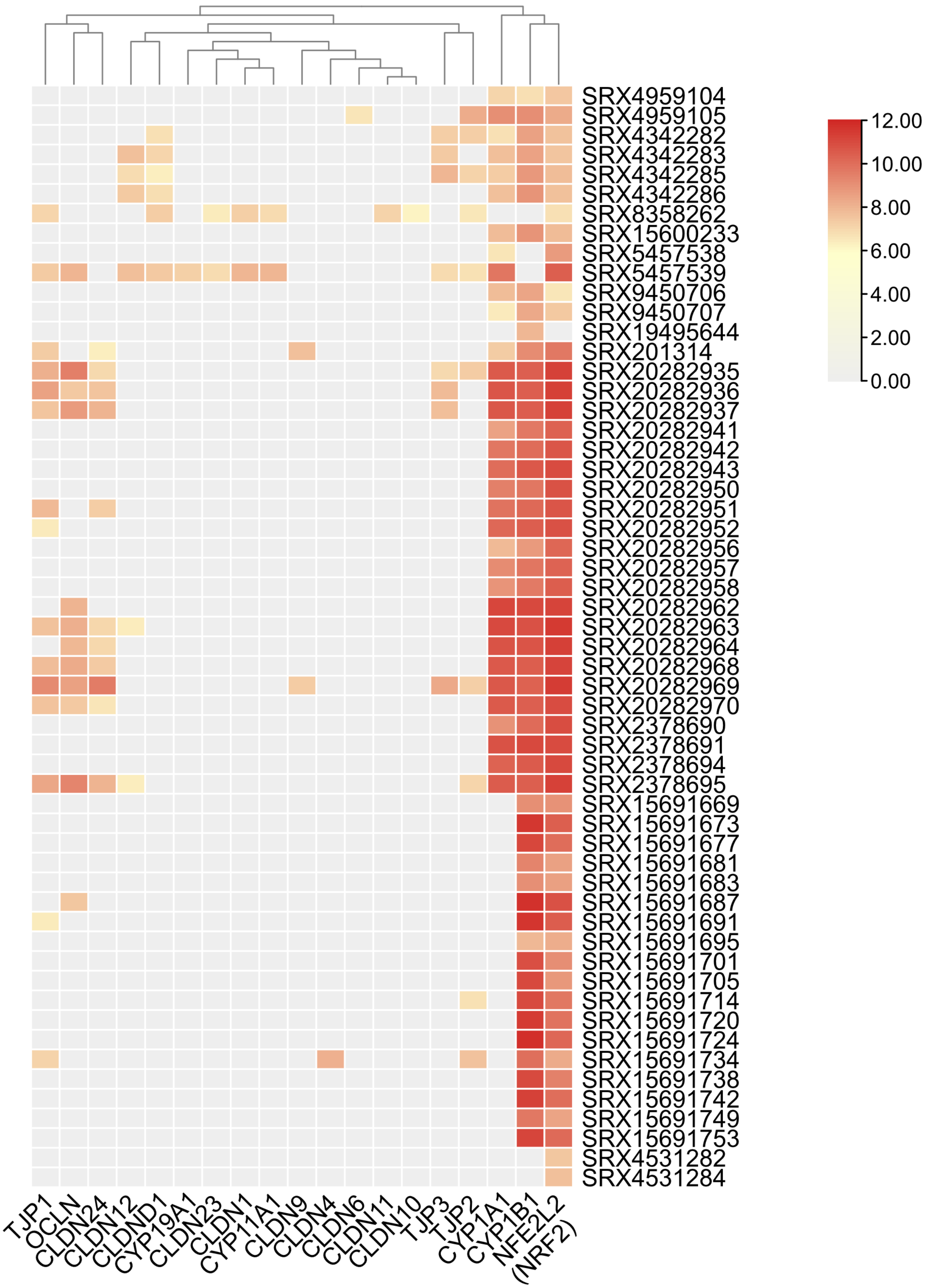

Figure S11. AHR-ligand ChIP-seq analysis.
Heatmap showing publicly available ChIP-Atlas (http://chip-atlas.org/target_genes) Chip-seq data of AHR ligand.

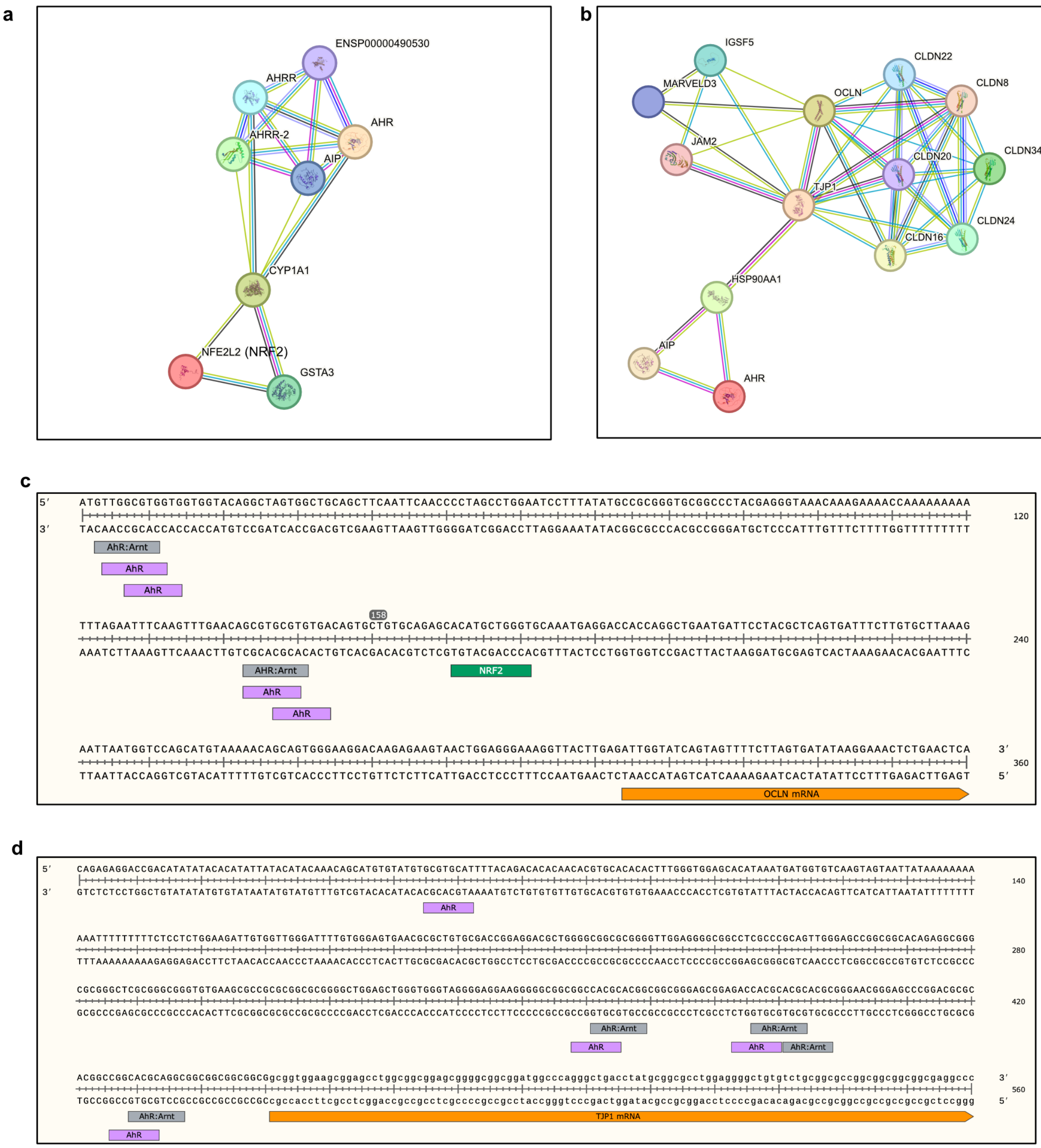

Figure S12. Interaction of AHR and target proteins.
a AHR targets NRF2 and CYP1A1 genes.
b AHR also targets tight junction proteins such as TJP1 and OCLN.
c AHR and NRF2 are predicted to bind promoter of OCLN gene.
d AHR is predicted to bind promoter of TJP1 gene.
